# Author Correction: Blue organic light-emitting diode with a turn-on voltage of 1.47V

**DOI:** 10.1038/s41467-026-72307-w

**Published:** 2026-04-24

**Authors:** Seiichiro Izawa, Masahiro Morimoto, Keisuke Fujimoto, Koki Banno, Yutaka Majima, Masaki Takahashi, Shigeki Naka, Masahiro Hiramoto

**Affiliations:** 1 Laboratory for Materials and Structures, Institute of Innovative Research, Tokyo Institute of Technology, 4259 Nagatsuta-cho, Midori-ku, Yokohama, Kanagawa 226-8503 Japan; 2 Joining and Welding Research Institute, Osaka University, 11-1, Mihogaoka, Ibaraki, Osaka, 567-0047 Japan; 3 Precursory Research for Embryonic Science and Technology (PRESTO), Japan Science and Technology Agency (JST), 4-1-8 Honcho, Kawaguchi, Saitama, 332-0012 Japan; 4 Academic Assembly Faculty of Engineering, University of Toyama, 3190 Gofuku, Toyama, 930-8555 Japan; 5 Department of Applied Chemistry, Faculty of Engineering, Shizuoka University, 3-5-1 Johoku, Naka-ku, Hamamatsu, Shizuoka, 432-8561 Japan; 6 Institute for Molecular Science, 5-1 Higashiyama, Myodaiji, Okazaki, Aichi 444-8787 Japan

**Keywords:** Organic LEDs, Organic LEDs

Correction to: *Nature Communications* 10.1038/s41467-023-41208-7, published online 20 September 2023

The authors would like to correct an error identified in the calculation of the external quantum efficiency (EQE). Specifically, the error originates from the choice of wavelength interval (nm) used in the numerical integration of spectral data. In the original manuscript, the wavelength interval was taken to be 1.4 nm, corresponding to the stated instrumental spectral resolution of the spectrometer. However, the actual wavelength interval of the acquired spectral data was 0.57–0.60 nm. After re-evaluating the calculation using this data interval as the wavelength interval, the authors found that the corrected values are approximately three-sevenths of those reported previously. The corrected calculation procedure was quantitatively validated by comparison with independently calibrated measurements obtained using an EQE measurement system based on an integrating sphere.

The authors are therefore replacing the original Fig. 2e and Fig. 4f with new plots, which are based on the verified procedure. Values in the last paragraph of the Results are similarly updated to read “…maximum EQE of the UC-OLED is 1.37%,” rather than the original “3.25%.”

It is important to note that this correction applies only to the absolute values. All relative comparisons between materials and experimental conditions remain unchanged, as they are scaled uniformly. The corrected maximum EQE of the blue upconversion (UC)-organic light-emitting diode (OLED) in Table S2 in the Supplementary Information is 1.37%. This correction does not affect the main claim of the original paper, the ultralow voltage turn-on at 1.47 V for blue emission, which is the lowest operating voltage thus far among any type of blue LEDs.

Additionally, the authors would like to correct an error in the *y*-axis label of Fig. S15c. The original label “EQE” in Fig. S15 now reads “IPCE.”

The authors apologize for any confusion this may have caused and state that the scientific conclusions of the original article are unaffected. Text, figures and Supplementary Information are now amended in the HTML and PDF versions of the article.

