# Supplementary material for: Blue organic light-emitting diode with a turn-on voltage of 1.47 V
Source: Nat Commun. 2023 Sep 20;14:5494. doi: 10.1038/s41467-023-41208-7 (PMC10511415; doi:10.1038/s41467-023-41208-7)
Supplement: Supplementary file 1 — Supplementary Information [file 41467_2023_41208_MOESM1_ESM.pdf]

Supplementary Information for

Blue Organic Light-Emitting Diode with a Turn-on Voltage of 1.47 V

Seiichiro Izawa[1,2,3*], Masahiro Morimoto[4*], Keisuke Fujimoto[5*], Koki Banno[5], Yutaka Majima[1], Masaki Takahashi[5], Shigeki Naka[4], and Masahiro Hiramoto[6]

[1]Laboratory for Materials and Structures, Tokyo Institute of Technology, 4259 Nagatsuta-cho, Midori-ku, Yokohama, Kanagawa 226-8503, Japan. [2]Joining and Welding Research Institute, Osaka University, 11-1, Mihogaoka, Ibaraki, Osaka, 567-0047, Japan, [3]Precursory Research for Embryonic Science and Technology (PRESTO), Japan Science and Technology Agency (JST), 4-1-8 Honcho, Kawaguchi, Saitama 332-0012, Japan. [4]Academic Assembly Faculty of Engineering, University of Toyama, 3190 Gofuku, Toyama 930-8555, Japan. [5]Department of Applied Chemistry, Faculty of Engineering, Shizuoka University, 3-5-1 Johoku, Naka-ku, Hamamatsu, Shizuoka, 432-8561, Japan. [6]Institute for Molecular Science, 5-1 Higashiyama, Myodaiji, Okazaki 444-8787, Aichi, Japan

E-Mail: izawa.s.ac@m.titech.ac.jp (S.I.), morimoto@eng.u-toyama.ac.jp (M.M.), fujimoto.keisuke@shizuoka.ac.jp (K.F.).

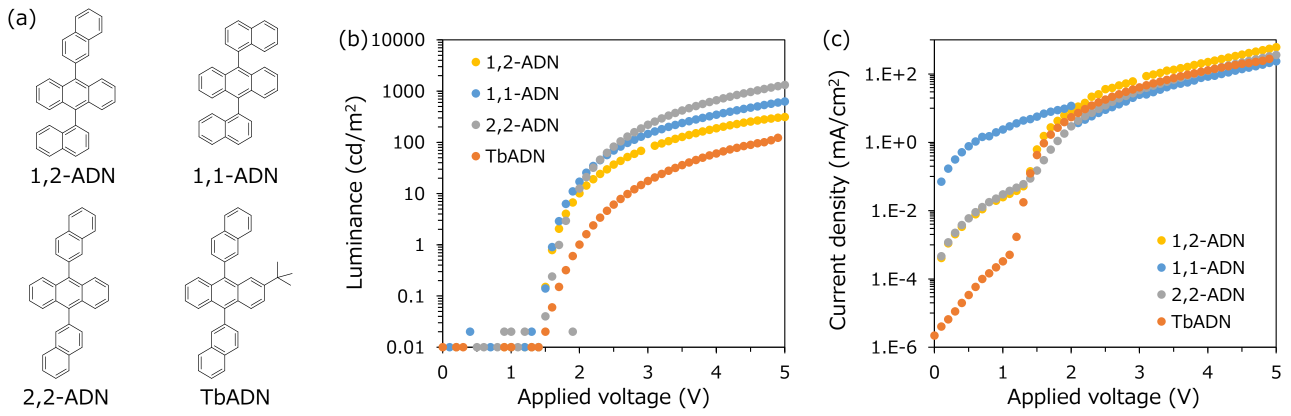

Figure S1. (a) Chemical structures of anthracene derivatives. (b) *L*–*V* and (c) *J*–*V* curves for the OLED devices with 1,2-ADN (yellow), 1,1-ADN (blue), 2,2-ADN (grey), or TbADN (orange) and NDI-HF.

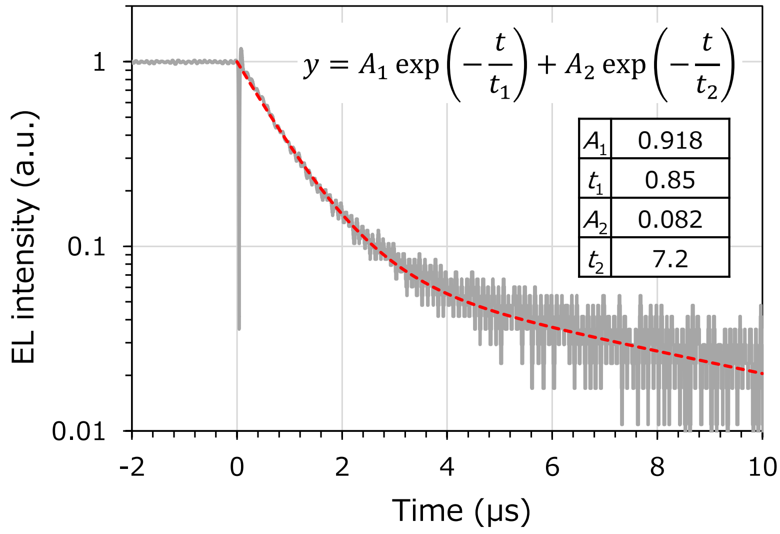

Figure S2. Decay curve of EL emission in 1,2-ADN/NDI-HF. The inset shows the fitted equation and the parameters.

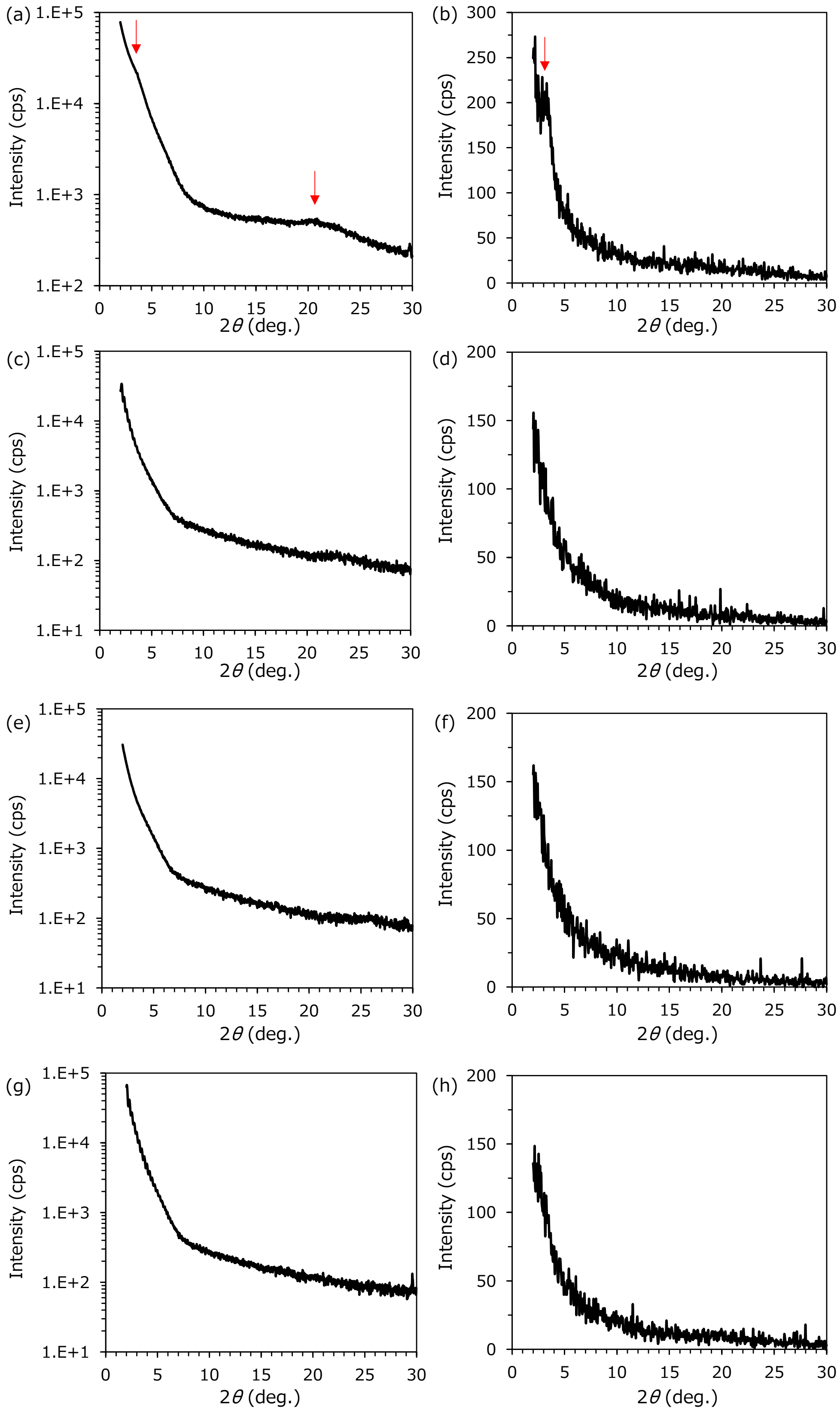

Figure S3. (a) Out-of-plane and (b) in-plane XRD patterns of the NDI-HF film on a silicon wafer. (c) Out-of-plane and (d) in-plane XRD patterns of the B4PYMPM film on a silicon wafer. (e) Out-of-

plane and (f) in-plane XRD patterns of the BPyOXD film on a silicon wafer. (g) Out-of-plane and (h) in-plane XRD patterns of the TmPyPB film on a silicon wafer.

Preparation of NDI derivatives

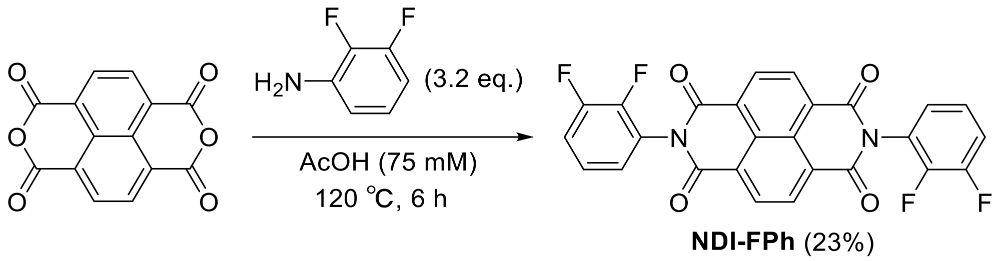

Synthesis of NDI-FPh. AcOH (10 mL, 75 mM) and 2,3-difluoroaniline (3.2 eq., 2.39 mmol, 0.24 mL) were added to a flask containing naphthalene-1,4,5,8-tetracarboxylic dianhydride (200 mg, 0.747 mmol) under argon atmosphere. The reaction mixture was stirred at 120°C for 6 h. After addition of water to the cooled reaction mixture, the resulting precipitates were collected by filtration and washed with water. The precipitates were purified by silica gel column chromatography eluting with AcOEt/$CHCl_3$ (volume ratio: 1/30). Recrystallization from $CHCl_3$/hexane gave **NDI-FPh** (83 mg, 0.170 mmol, 23%).

Compound data of NDI-FPh. ^{1}H NMR (300 MHz, $CDCl_3$) δ 8.87 (s, 4H), 7.45–7.30 (m, 4H), 7.24–7.14 (m, 2H); ^{13}C NMR (75 MHz, DMSO-d_6) δ 159.5, 131.7, 128.4, 124.7. Several signals were not observed due to the low solubility and complicated spin-spin coupling with ^{19}F nucleus. HR-ESI-MS: m/z = 471.0556 $[M - F]^+$, calc. for $C_{26}H_{10}F_3N_2O_4$: 471.0593.

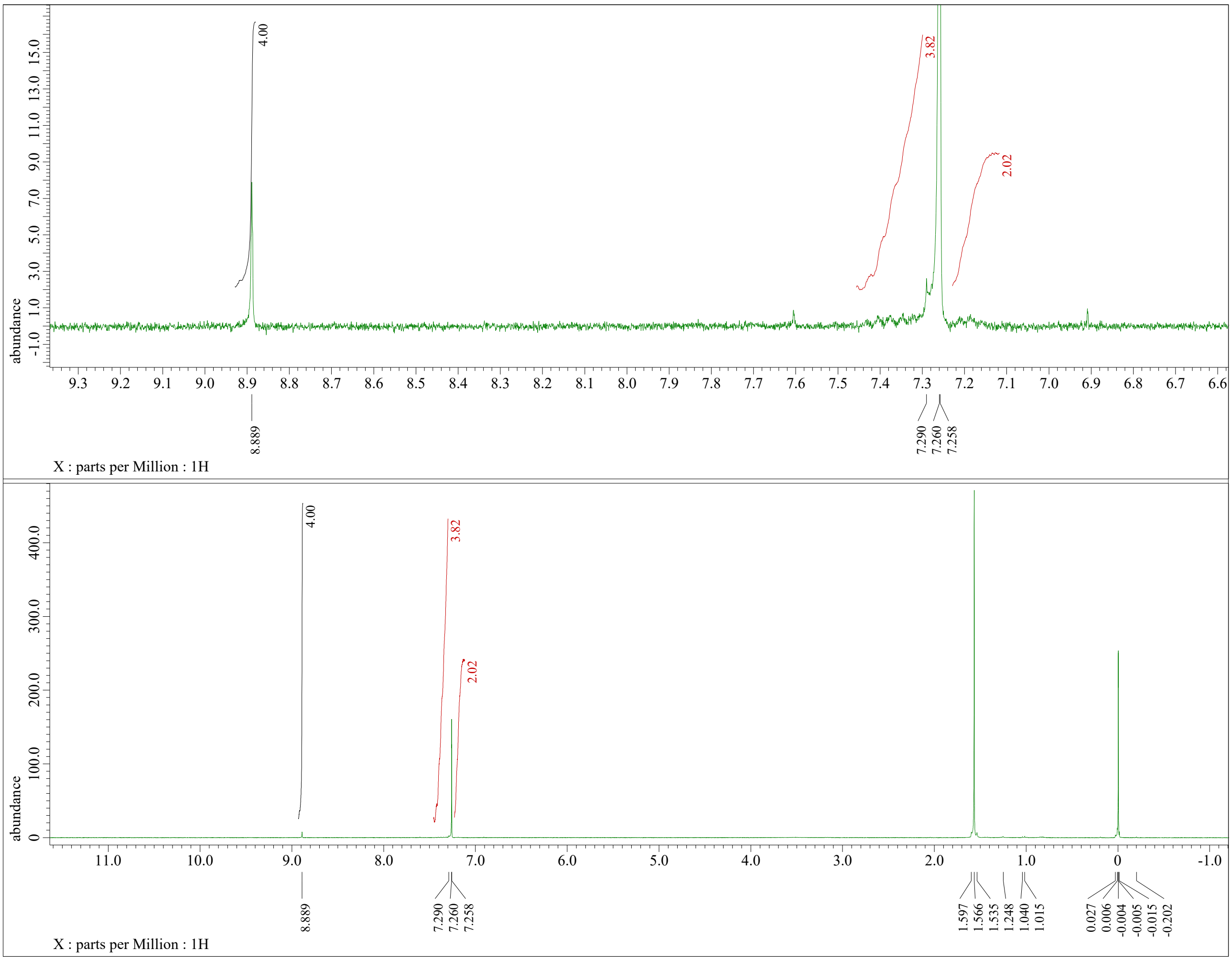

Figure S4. ^{1}H NMR spectrum of **NDI-FPh** in $CDCl_3$ at 25°C.

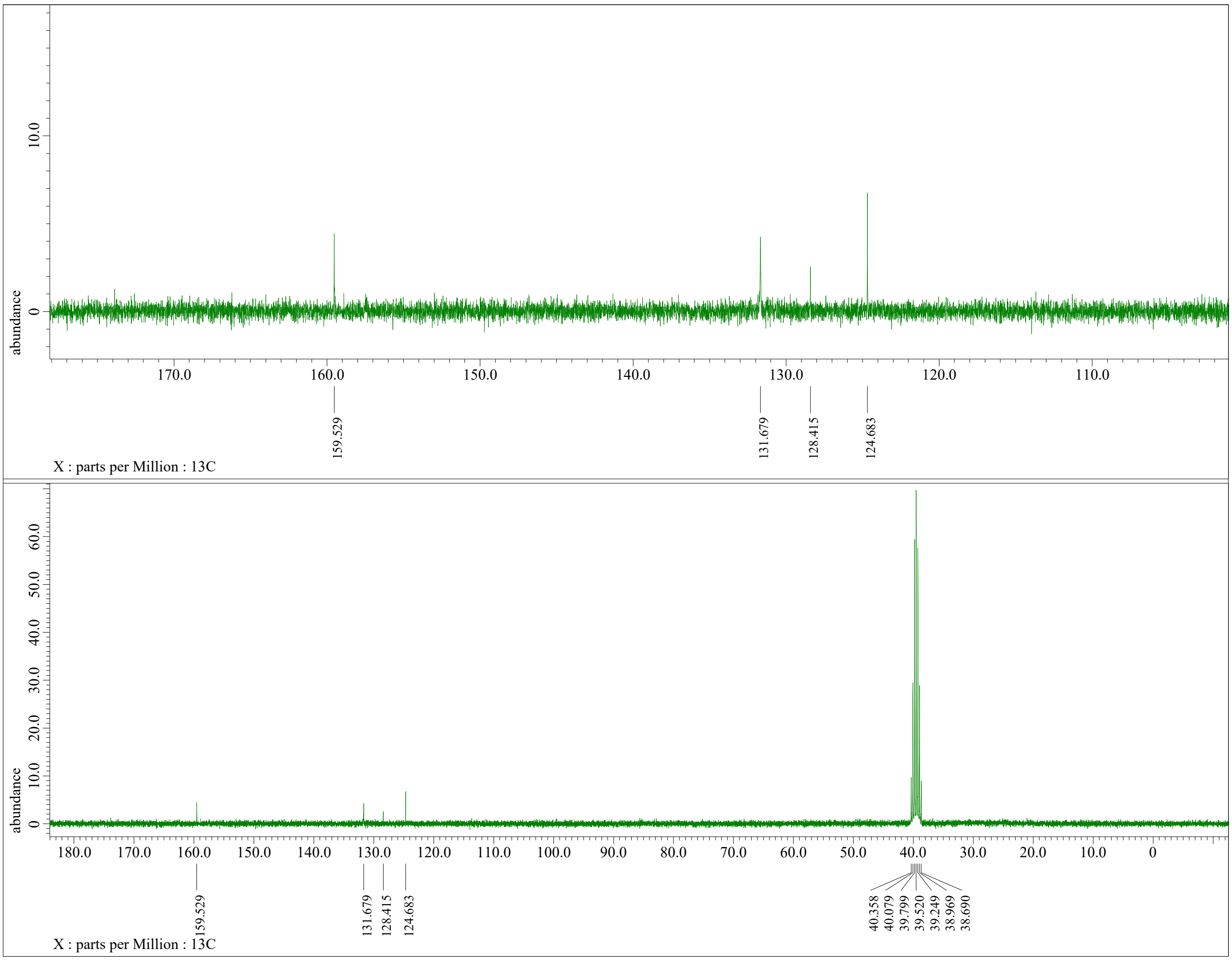

Figure S5. ^{13}C NMR spectrum of **NDI-FPh** in DMSO-d_6 at 25°C.

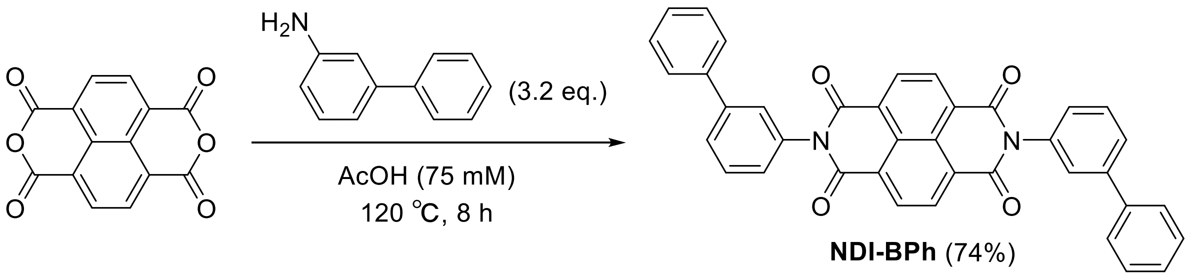

Synthesis of NDI-BPh. AcOH (10 mL, 75 mM) was added to a flask containing naphthalene-1,4,5,8-tetracarboxylic dianhydride (204 mg, 0.749 mmol) and 3-aminobiphenyl (3.2 eq., 2.37 mmol, 401 mg) under argon atmosphere. The reaction mixture was stirred at 120°C for 4 h. After addition of water to the cooled reaction mixture, the resulting precipitates were collected by filtration and washed with water. The precipitates were purified by passing through short silica gel column eluting with AcOEt/$CHCl_3$ (volume ratio: 1/15). Recrystallization from $CHCl_3$/MeOH gave **NDI-BPh** (316 mg, 0.554 mmol, 74%).

Compound data of NDI-BPh. ^{1}H NMR (300 MHz, $CDCl_3$) δ 8.88 (s, 4H), 7.78 (d, J = 7.5 Hz, 2H), 7.70–7.63 (m, 6H), 7.57 (s, 2H), 7.45 (t, J = 7.5 Hz, 4H), 7.40–7.32 (m, 4H); ^{13}C NMR (75 MHz, $CDCl_3$) δ 163.1, 143.1, 140.2, 131.7, 130.1, 129.0, 128.2, 127.5, 127.4, 127.2. Several signals were not observed due to the signal overlapping. HR-ESI-MS: m/z = 571.1650 [M + H]$^{+}$, calc. for $C_{38}H_{23}N_2O_4$: 571.1658.

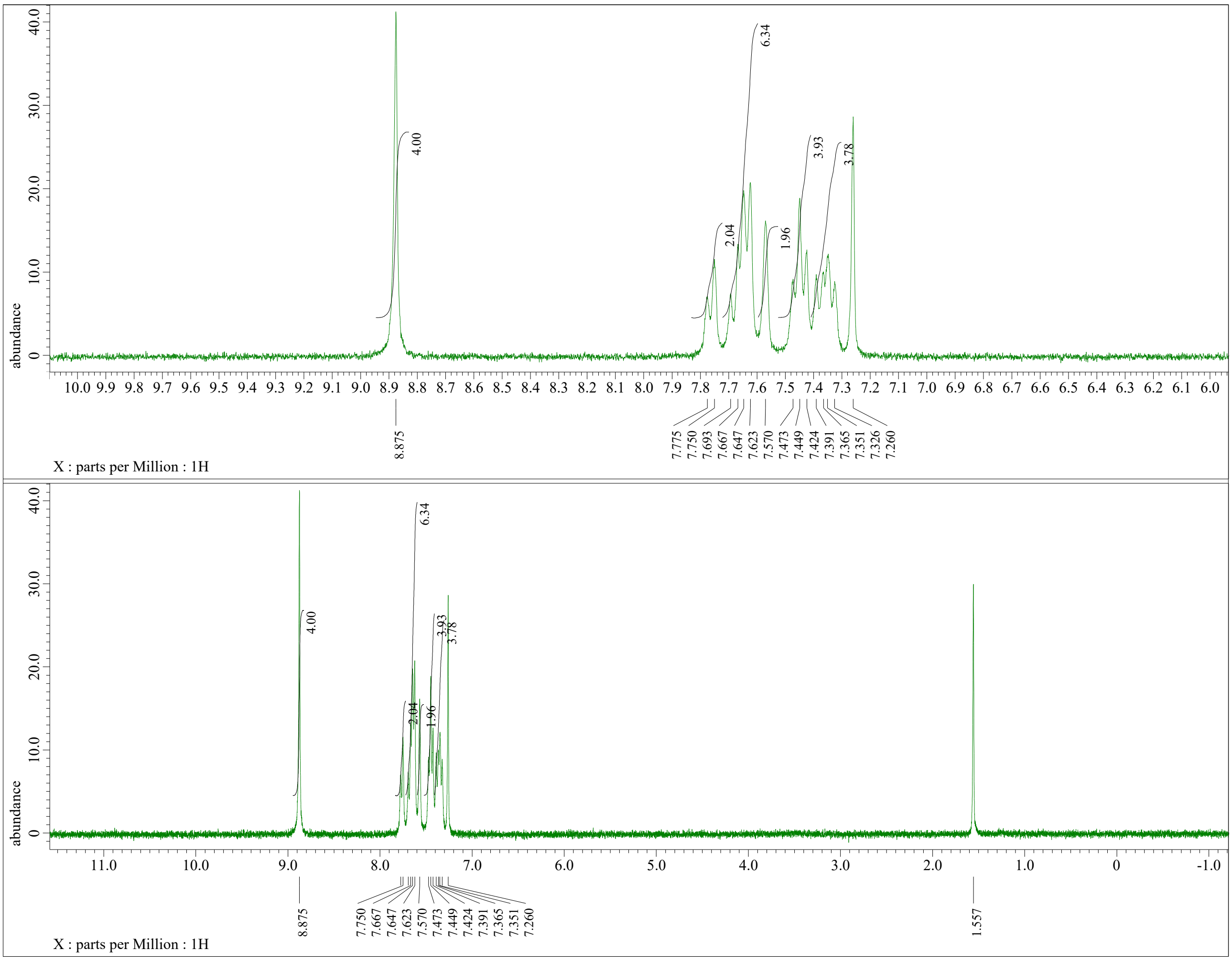

Figure S6. ^{1}H NMR spectrum of **NDI-BPh** in $CDCl_3$ at 25°C.

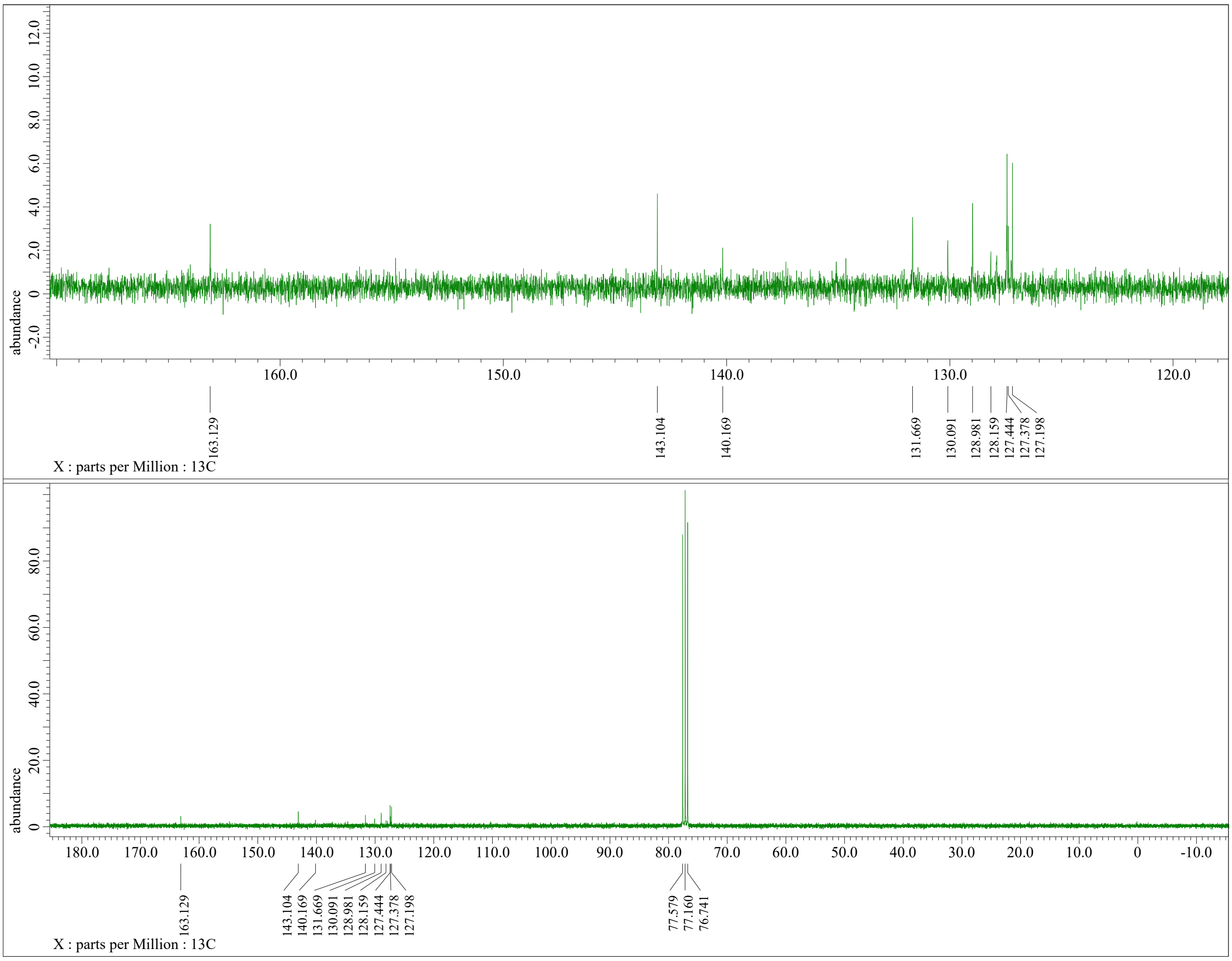

Figure S7. ^{13}C NMR spectrum of **NDI-BPh** in $CDCl_3$ at 25°C.

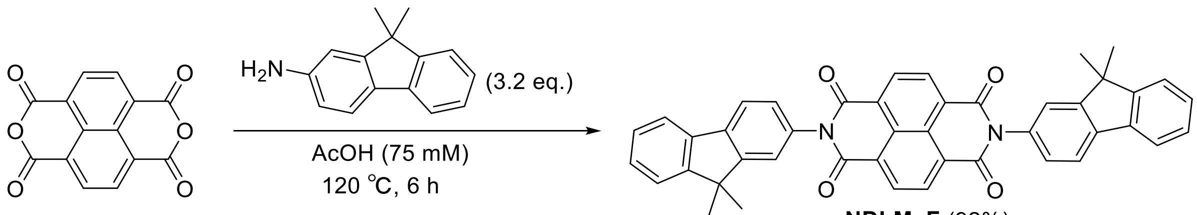

Synthesis of NDI-MeF. AcOH (10 mL, 75 mM) was added to a flask containing naphthalene-1,4,5,8-tetracarboxylic dianhydride (199 mg, 0.747 mmol) and 2-amino-9,9-dimethylfluorene (3.2 eq., 2.40 mmol, 503 mg) under argon atmosphere. The reaction mixture was stirred at 120°C for 6 h. After addition of water to the cooled reaction mixture, the resulting precipitates were collected by filtration and washed with water. The precipitates were purified by silica gel column chromatography eluting with AcOEt/CH_2Cl_2 (volume ratio: 1/15). Recrystallization from CH_2Cl_2/MeOH gave **NDI-MeF** (448 mg, 0.688 mmol, 92%).

Compound data of NDI-MeF. ^{1}H NMR (300 MHz, $CDCl_3$) δ 8.88 (s, 4H), 7.92 (d, J = 8.1 Hz, 4H), 7.81–7.78 (m, 2H), 7.50–7.46 (d, J = 6.0 Hz, 2H), 7.42–7.36 (m, 6H), 7.32 (dd, J = 7.8 and 1.8 Hz, 2H), 1.56 (s, 12H); ^{13}C NMR (75 MHz, $CDCl_3$) δ 164.1, 155.8, 154.8, 141.2, 139.2, 134.2, 132.4, 128.1, 128.0, 123.6, 121.7, 121.3, 48.1, 28.0. Several signals were not observed due to the signal overlapping. HR-ESI-MS: m/z = 651.2265 $[M + H]^+$, calc. for $C_{44}H_{31}N_2O_4$: 651.2284.

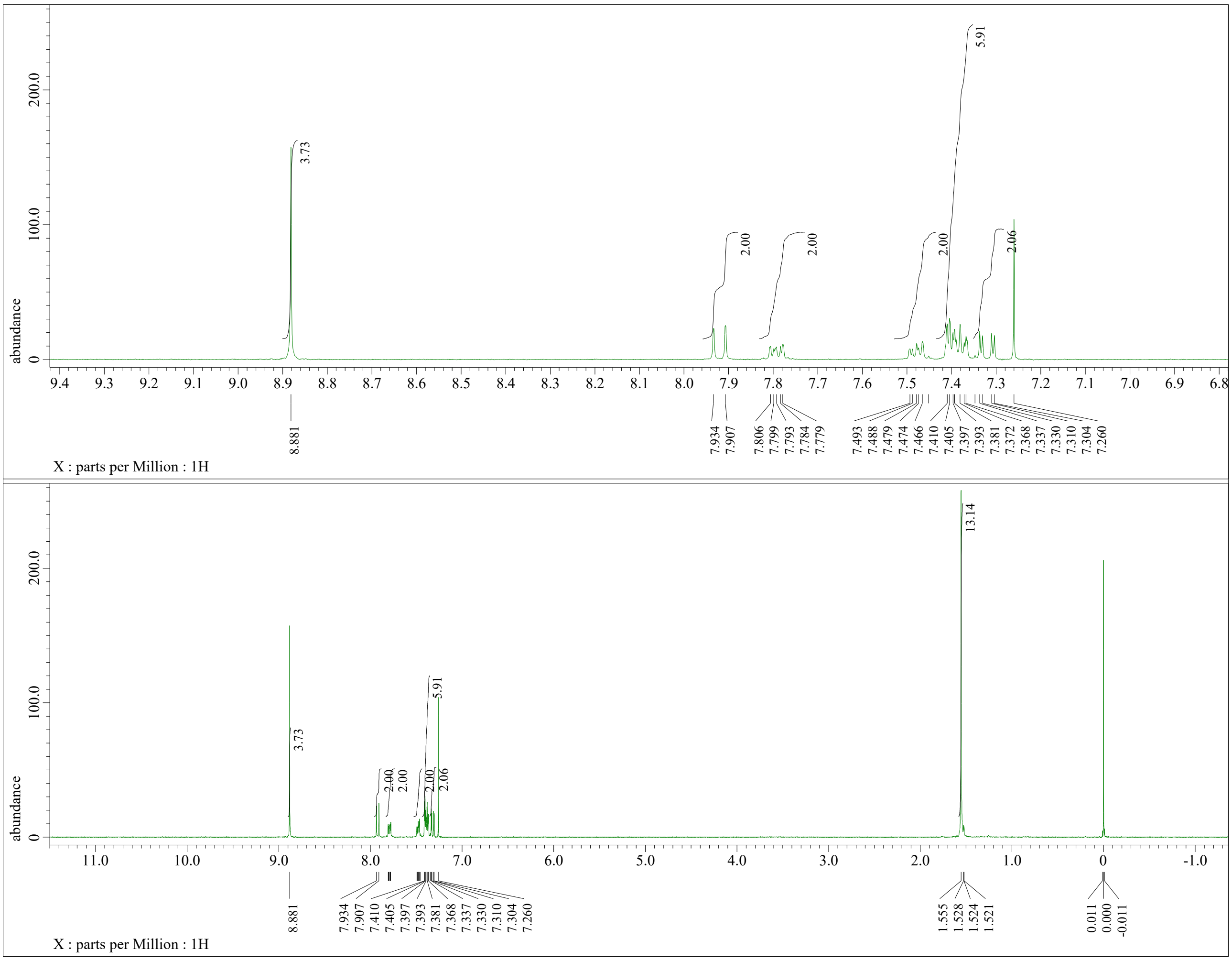

Figure S8. ^{1}H NMR spectrum of **NDI-MeF** in $CDCl_3$ at 25°C.

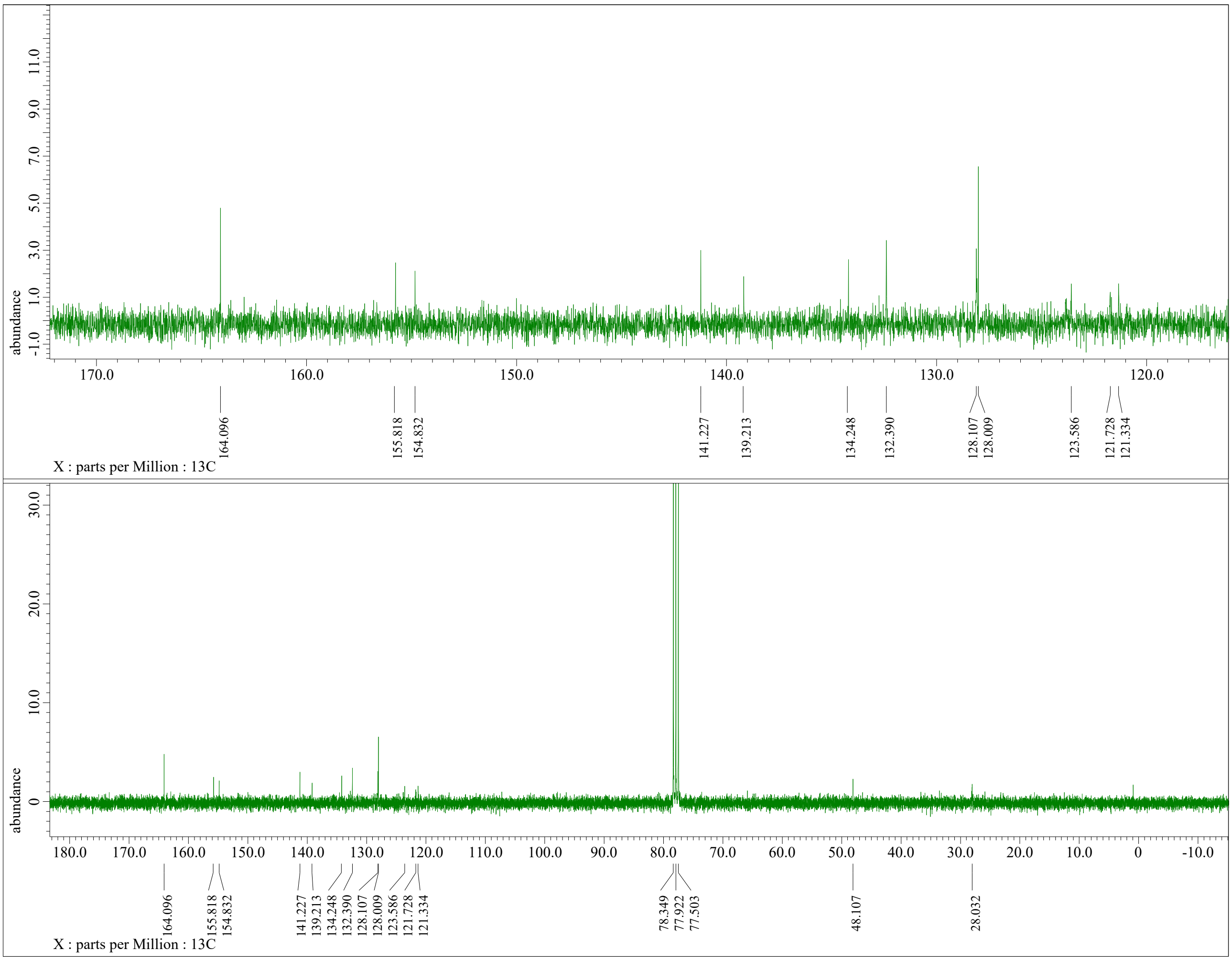

Figure S9. ^{13}C NMR spectrum of **NDI-MeF** in $CDCl_3$ at 25°C.

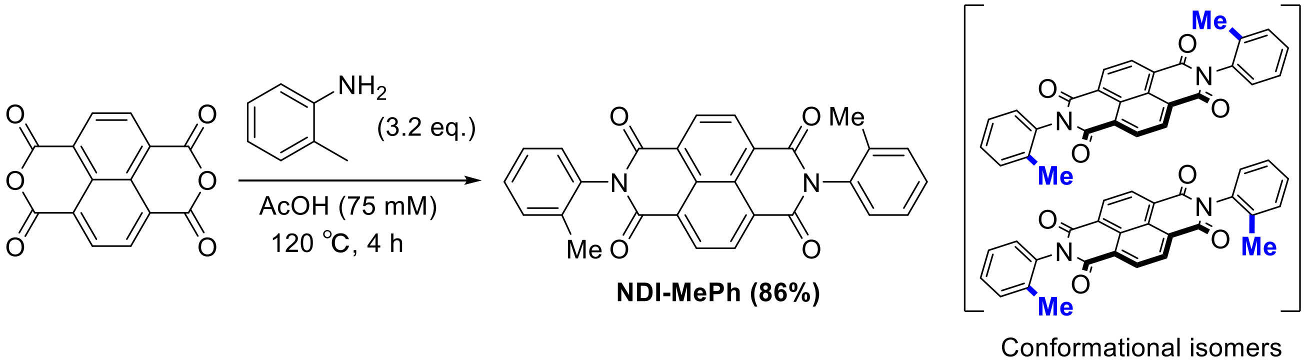

Synthesis of NDI-MePh. AcOH (30 mL, 75 mM) and *o*-toluidine (3.2 eq., 7.17 mmol, 0.77 mL) were added to a flask containing naphthalene-1,4,5,8-tetracarboxylic dianhydride (600 mg, 2.24 mmol) under argon atmosphere. The reaction mixture was stirred at 120°C for 4 h. After addition of water to the cooled reaction mixture, the resulting precipitates were collected by filtration and washed with water and MeOH. The precipitates were purified by passing through short silica gel column eluting

with AcOEt/$CHCl_3$ (volume ratio: 1/8). Recrystallization from $CHCl_3$/MeOH gave **NDI-MePh** (855 mg, 1.92 mmol, 86%).

Compound data of NDI-MePh. ^1H NMR (300 MHz, $CDCl_3$) δ 8.87 (s, 4H), 7.46–7.40 (m, 6H), 7.25–7.22 (m, 2H), 2.21 (s, 3H), 2.20 (s, 3H). Two conformational isomers were independently observed in the ^1H NMR. ^{13}C NMR (75 MHz, $CDCl_3$) δ 162.5, 135.7, 135.7, 133.8, 131.5, 131.3, 129.6, 128.3, 127.4, 127.0, 17.6, 17.6; HR-ESI-MS: m/z = 469.1145 $[M + Na]^+$, calc. for $C_{28}H_{18}N_2O_4Na$: 469.1164.

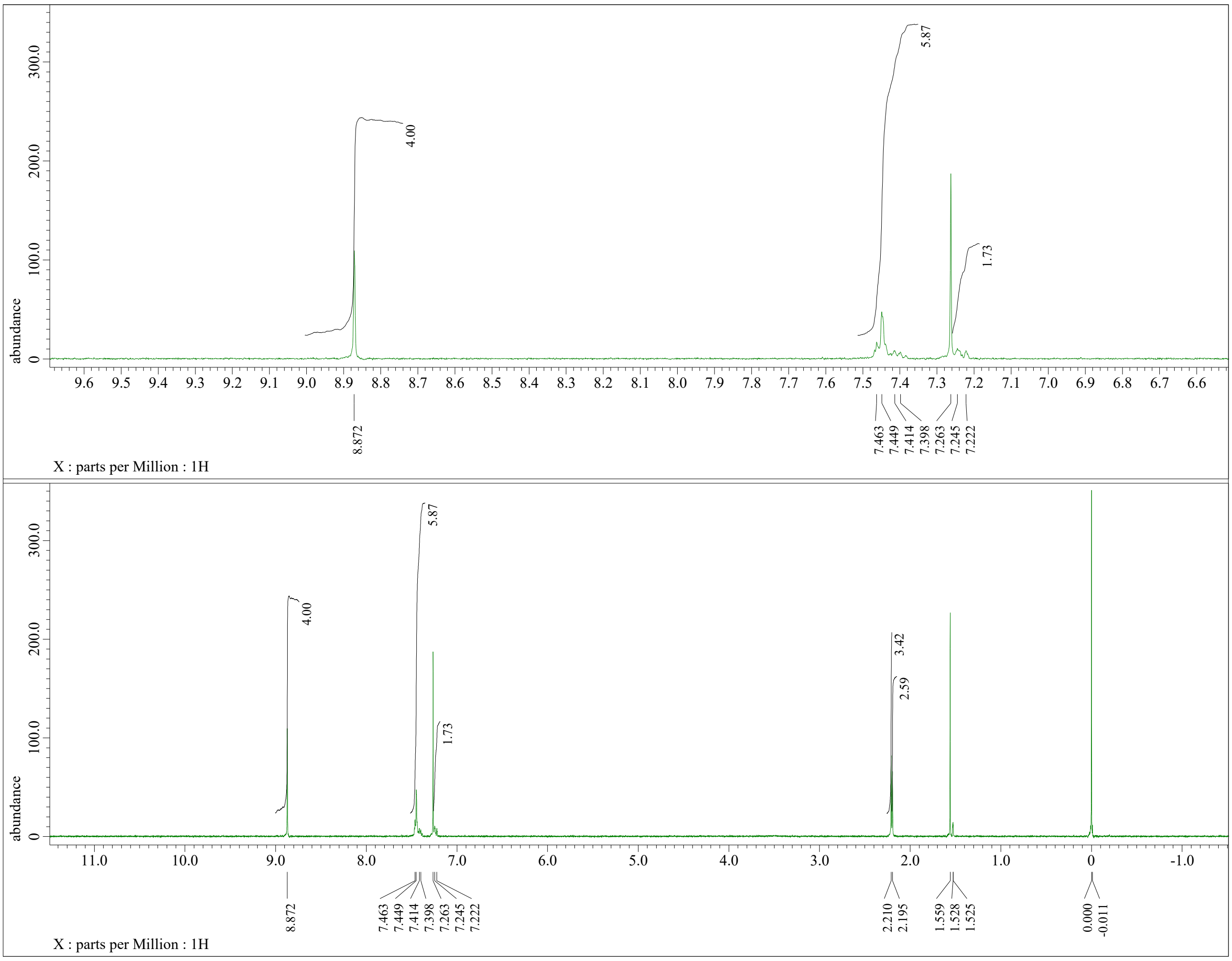

Figure S10. ^1H NMR spectrum of **NDI-MePh** in $CDCl_3$ at 25°C.

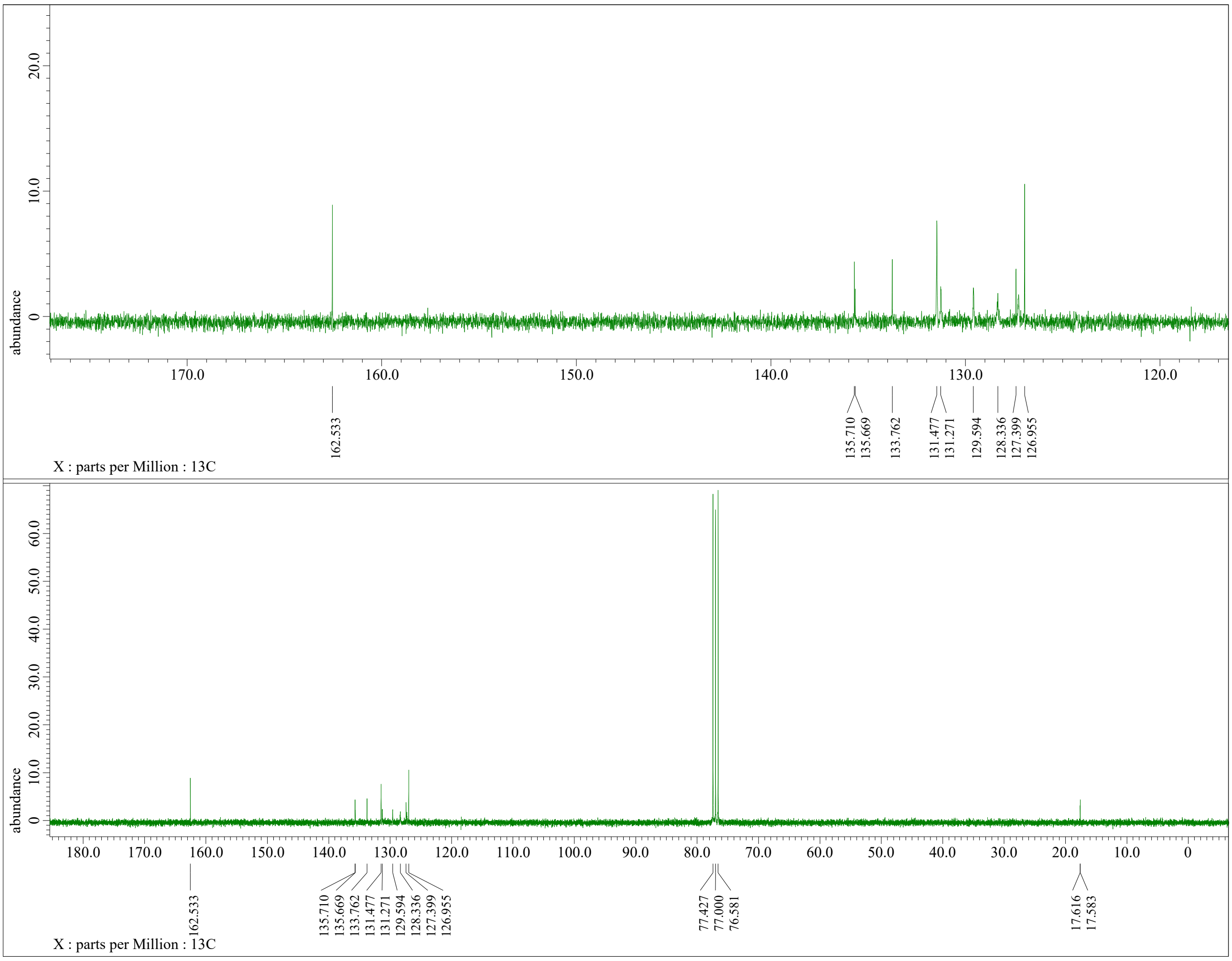

Figure S11. ^{13}C NMR spectrum of **NDI-MePh** in $CDCl_3$ at 25°C.

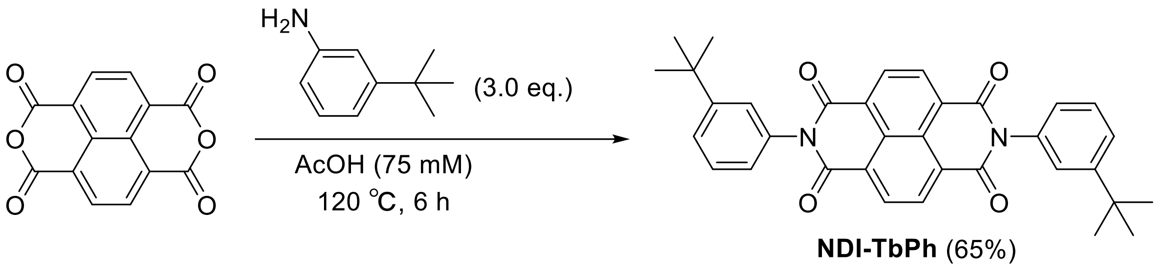

Synthesis of NDI-TbPh. AcOH (10 mL, 75 mM) and 3-*tert*-butylaniline (3.0 eq., 2.26 mmol, 0.35 mL) were added to a flask containing naphthalene-1,4,5,8-tetracarboxylic dianhydride (202 mg, 0.753 mmol) under argon atmosphere. The reaction mixture was stirred at 120°C for 8 h. After addition of water to the cooled reaction mixture, the resulting precipitates were collected by filtration and washed with water and MeOH. The precipitates were purified by passing through short silica gel column eluting with AcOEt/$CHCl_3$ (volume ratio: 1/30). Recrystallization from $CHCl_3$/MeOH gave **NDI-TbPh** (259 mg, 0.488 mmol, 65%).

Compound data of NDI-TbPh. ^{1}H NMR (300 MHz, $CDCl_3$) δ 8.85 (s, 4H), 7.58–7.50 (m, 4H), 7.33 (t, J = 1.8 Hz, 2H), 7.16 (dt, J = 7.2 and 1.8 Hz, 2H), 1.38 (s, 18H); ^{13}C NMR (75 MHz, $CDCl_3$) δ 163.0, 152.8, 134.2, 131.4, 129.1, 127.1, 127.0, 126.3, 125.3, 34.9, 31.3; HR-ESI-MS: m/z = 531.2271 $[M + H]^+$, calc. for $C_{34}H_{31}N_2O_4$: 531.2284.

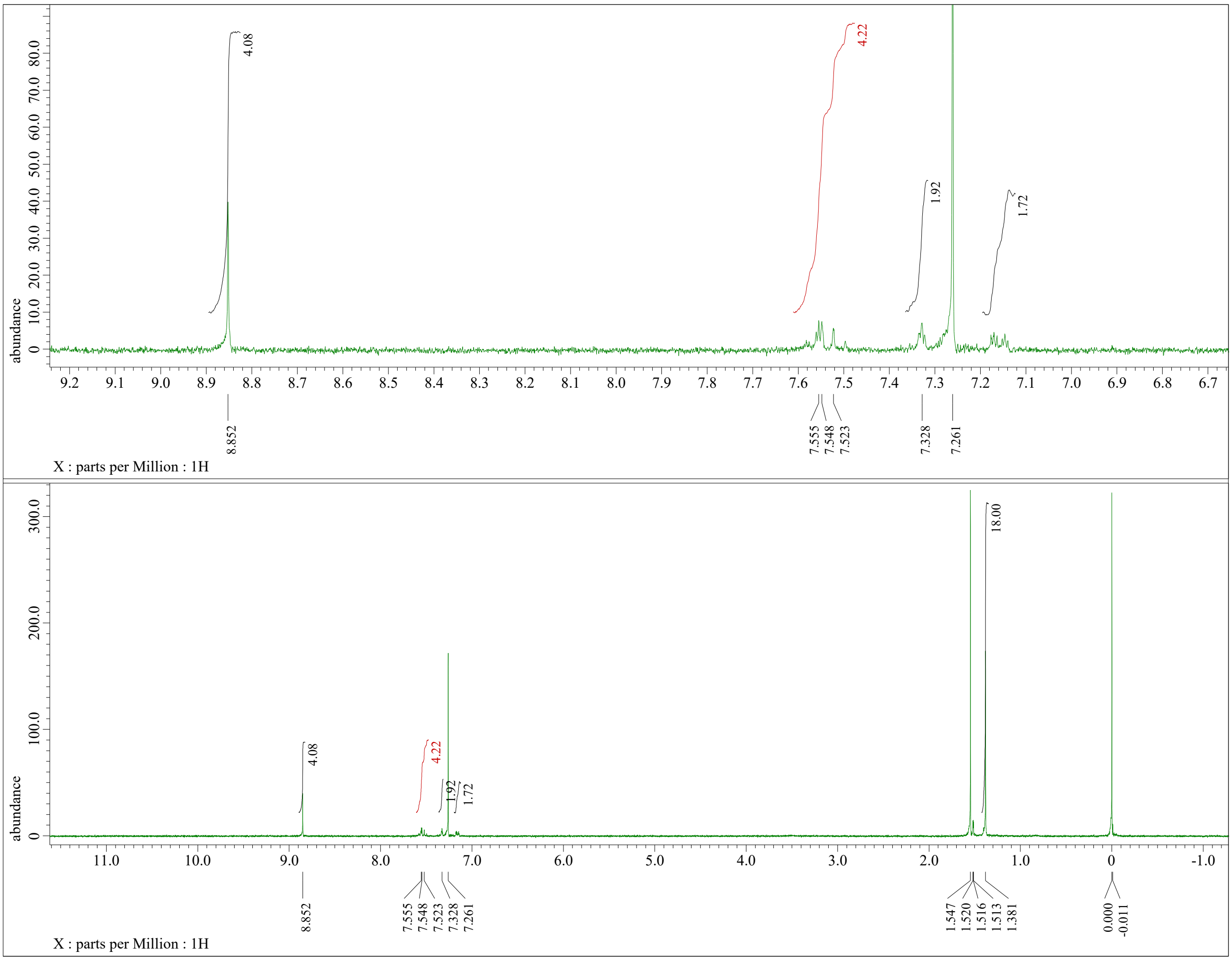

Figure S12. ^{1}H NMR spectrum of **NDI-TbPh** in $CDCl_3$ at 25°C.

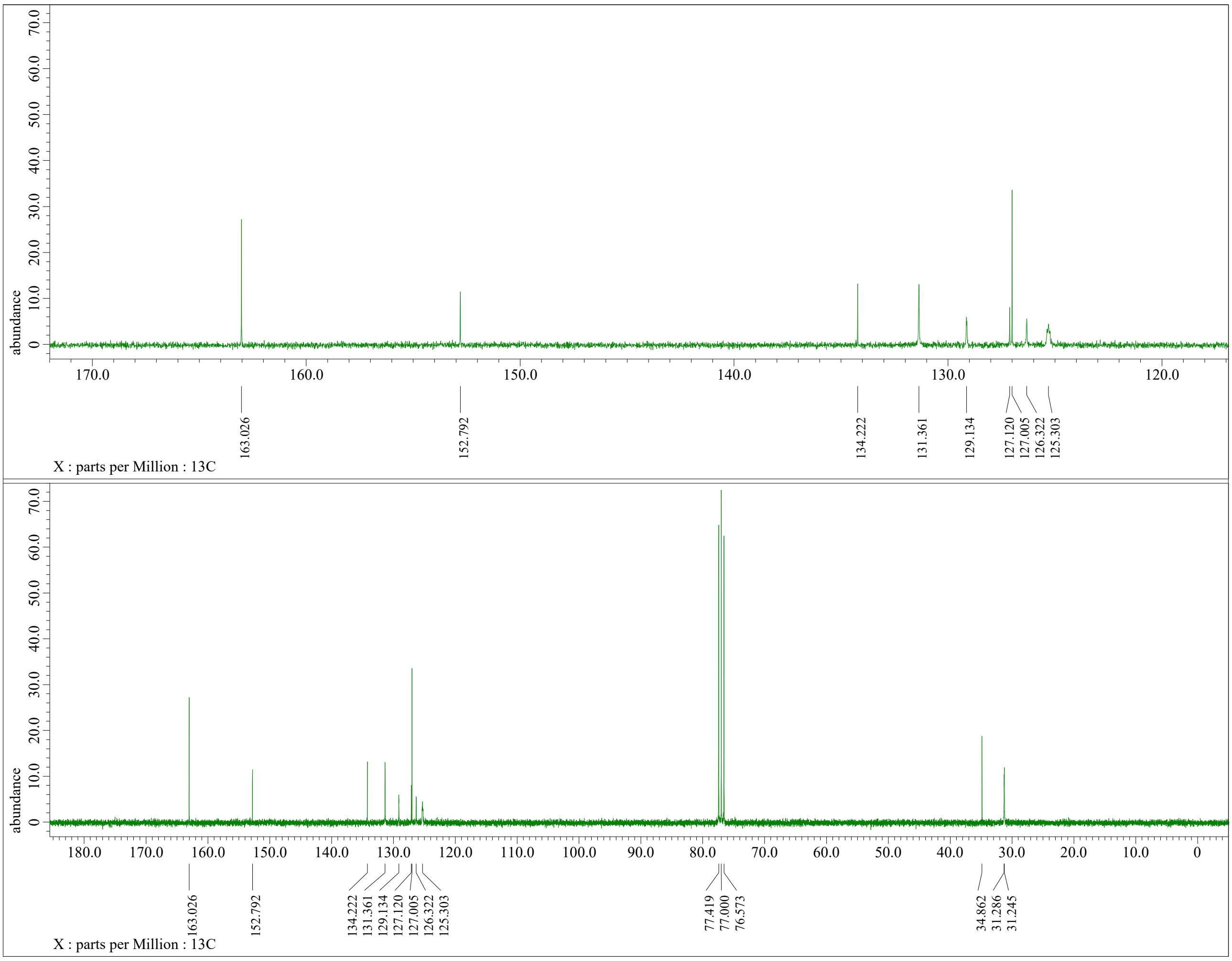

Figure S13. ^{13}C NMR spectrum of **NDI-TbPh** in $CDCl_3$ at 25°C.

Molecular Properties of NDI derivatives

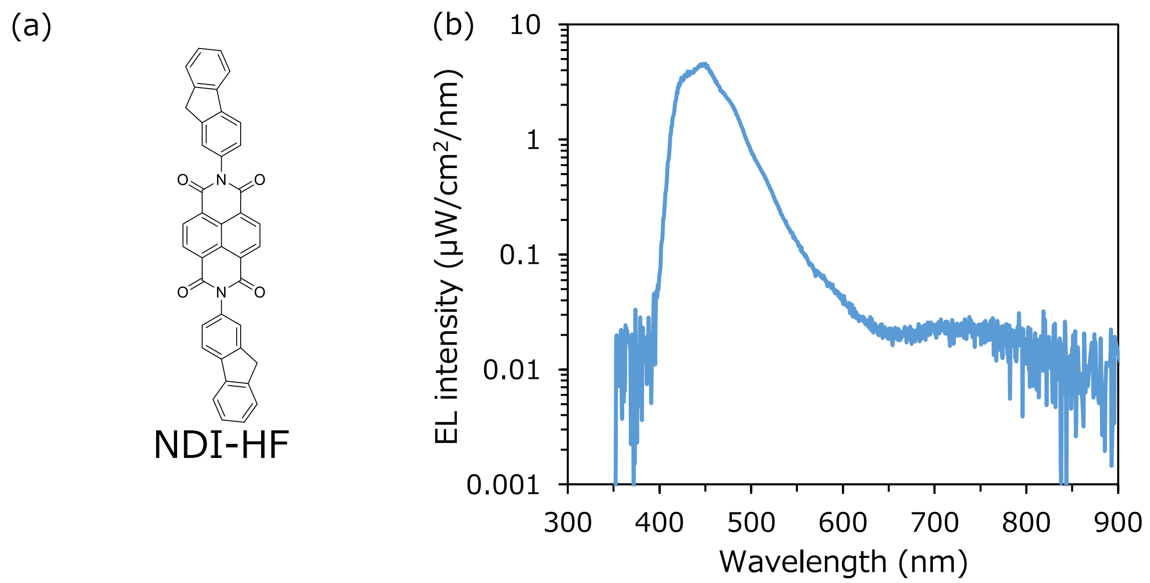

Figure S14. (a) Chemical structure of NDI-HF. (b) EL emission spectrum under a constant current flow (100 mA/cm^2).

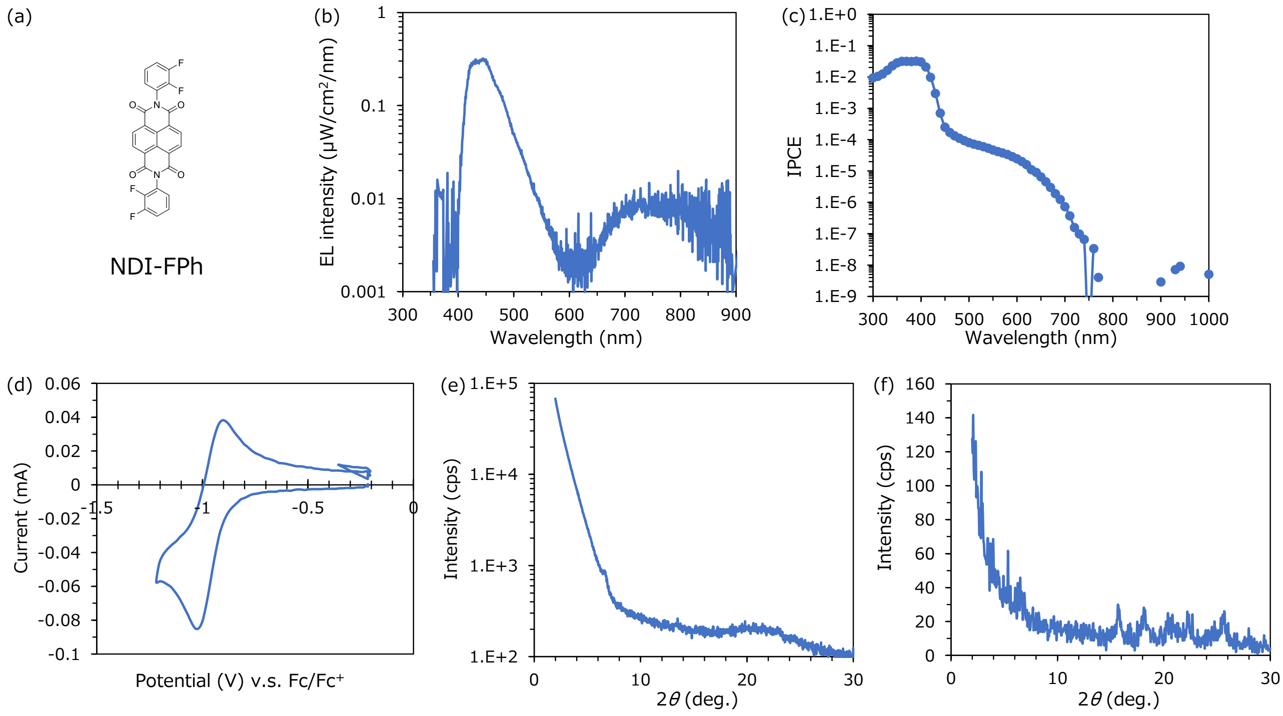

Figure S15. (a) Chemical structure of NDI-FPh. (b) EL emission spectrum under a constant current flow (100 mA/cm^2) and (c) highly sensitive IPCE spectra of the 1,2-ADN/NDI-FPh device. (d) Cyclic

voltammogram of NDI-FPh in solution. (e) Out-of-plane and (f) in-plane XRD patterns of the NDI-FPh film on a silicon wafer.

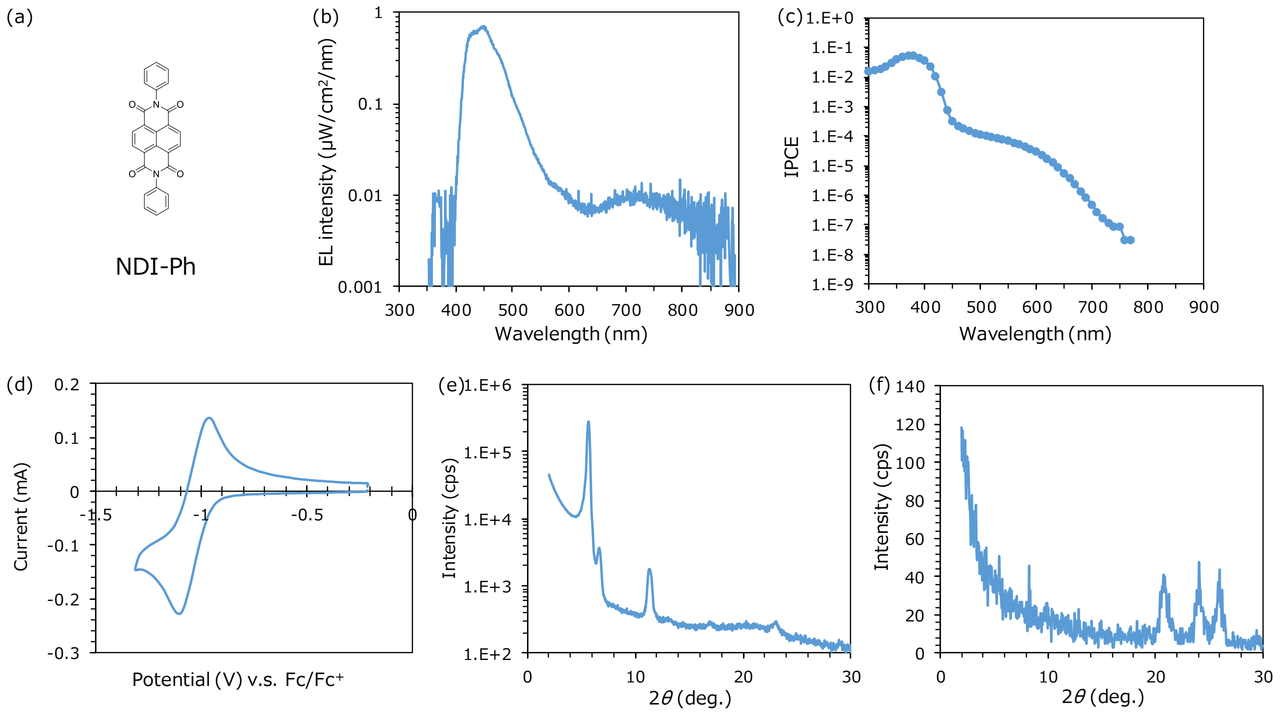

Figure S16. (a) Chemical structure of NDI-Ph. (b) EL emission spectrum under a constant current flow (100 mA/cm^2) and (c) highly sensitive IPCE spectra of the 1,2-ADN/NDI-Ph device. (d) Cyclic voltammogram of NDI-Ph in solution. (e) Out-of-plane and (f) in-plane XRD patterns of the NDI-Ph film on a silicon wafer.

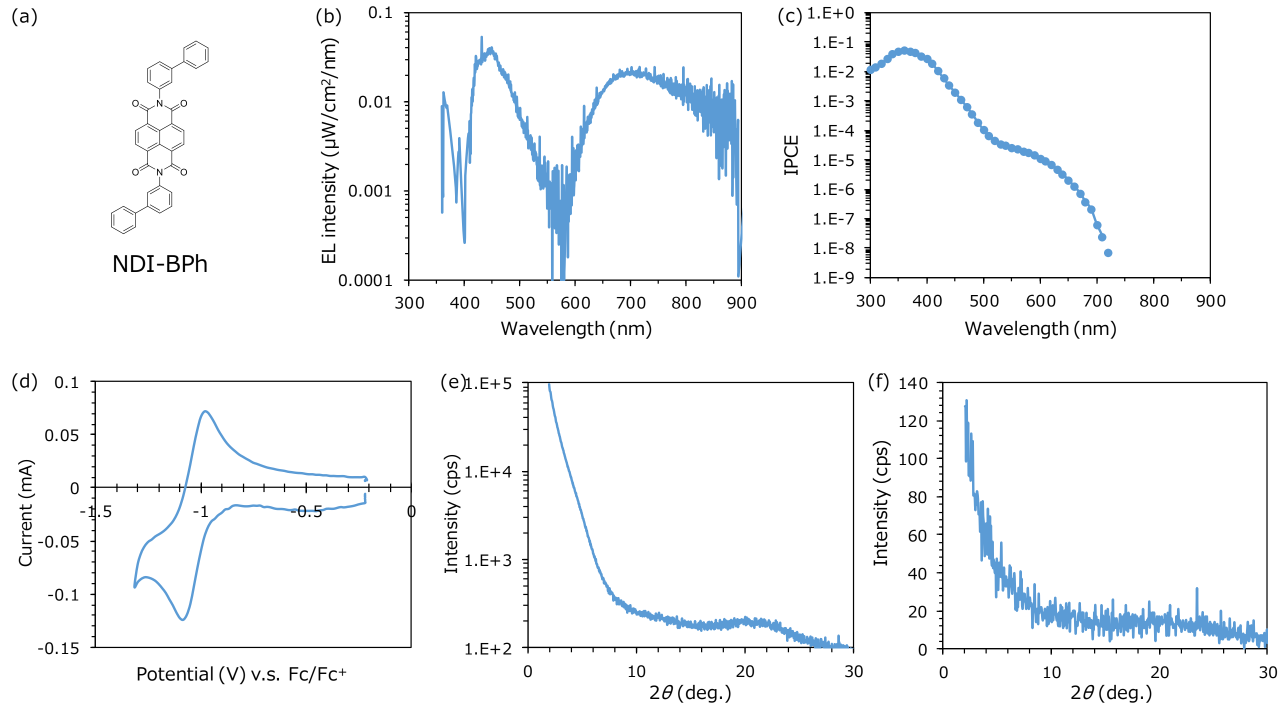

Figure S17. (a) Chemical structure of NDI-BPh. (b) EL emission spectrum under a constant current flow (100 mA/cm^2) and (c) highly sensitive IPCE spectra of the 1,2-ADN/NDI-BPh device. (d) Cyclic voltammogram of NDI-BPh in solution. (e) Out-of-plane and (f) in-plane XRD patterns of the NDI-BPh film on a silicon wafer.

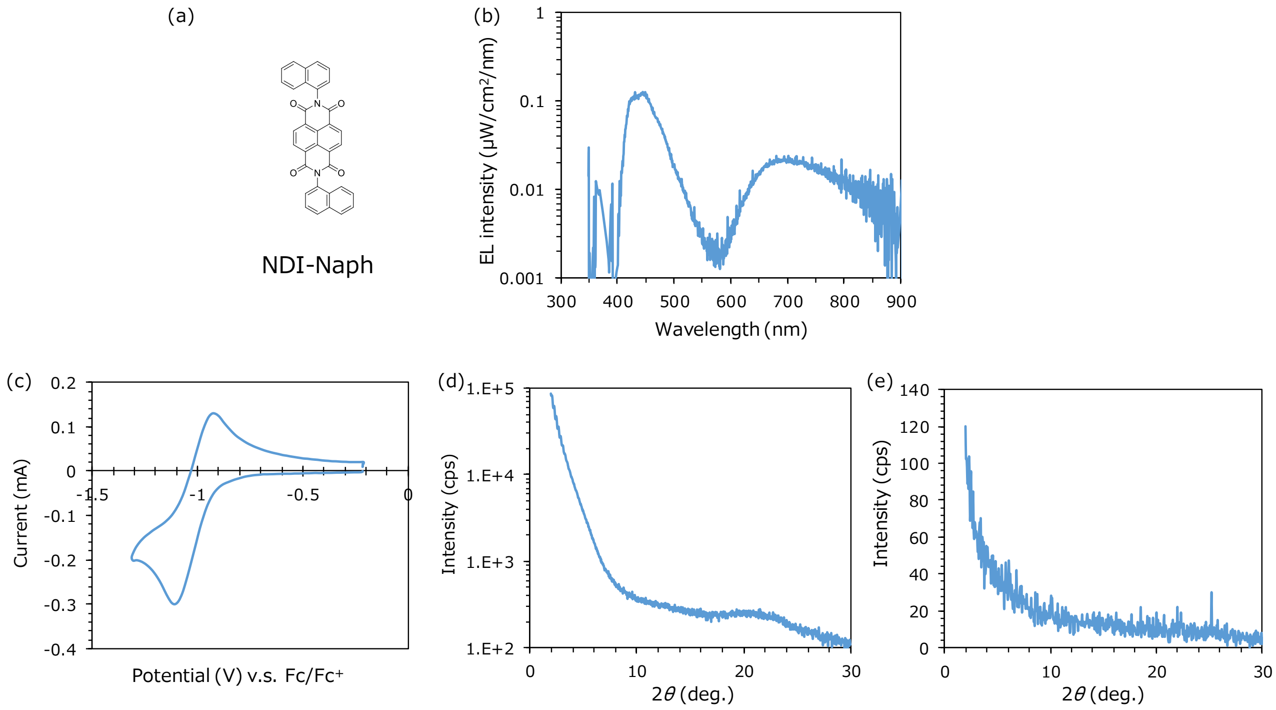

Figure S18. (a) Chemical structure of NDI-Naph. (b) EL emission spectrum under a constant current flow (100 mA/cm^2) of the 1,2-ADN/NDI-Naph device. (c) Cyclic voltammogram of NDI-Naph solution. (d) Out-of-plane and (e) in-plane XRD patterns of the NDI-Naph film on a silicon wafer.

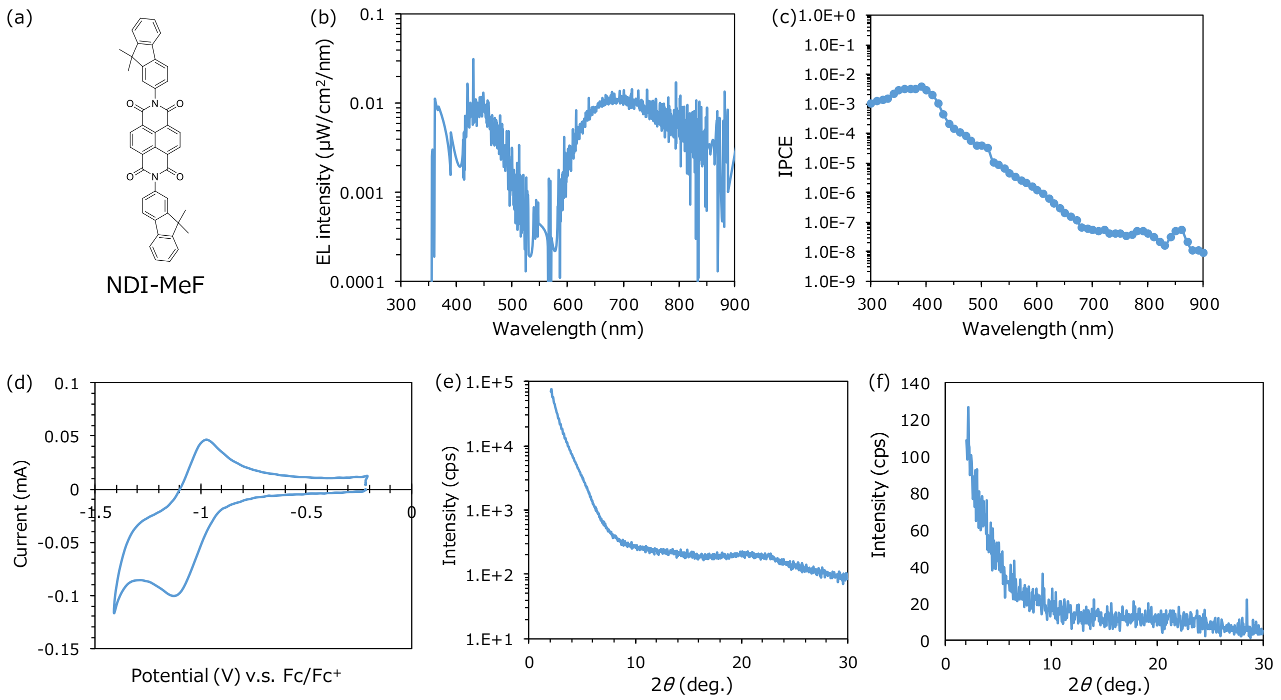

Figure S19. (a) Chemical structure of NDI-MeF. (b) EL emission spectrum under a constant current flow (100 mA/cm^2) and (c) highly sensitive IPCE spectra of the 1,2-ADN/NDI-MeF device. (d) Cyclic voltammogram of NDI-MeF in solution. (e) Out-of-plane and (f) in-plane XRD patterns of the NDI-MeF film on a silicon wafer.

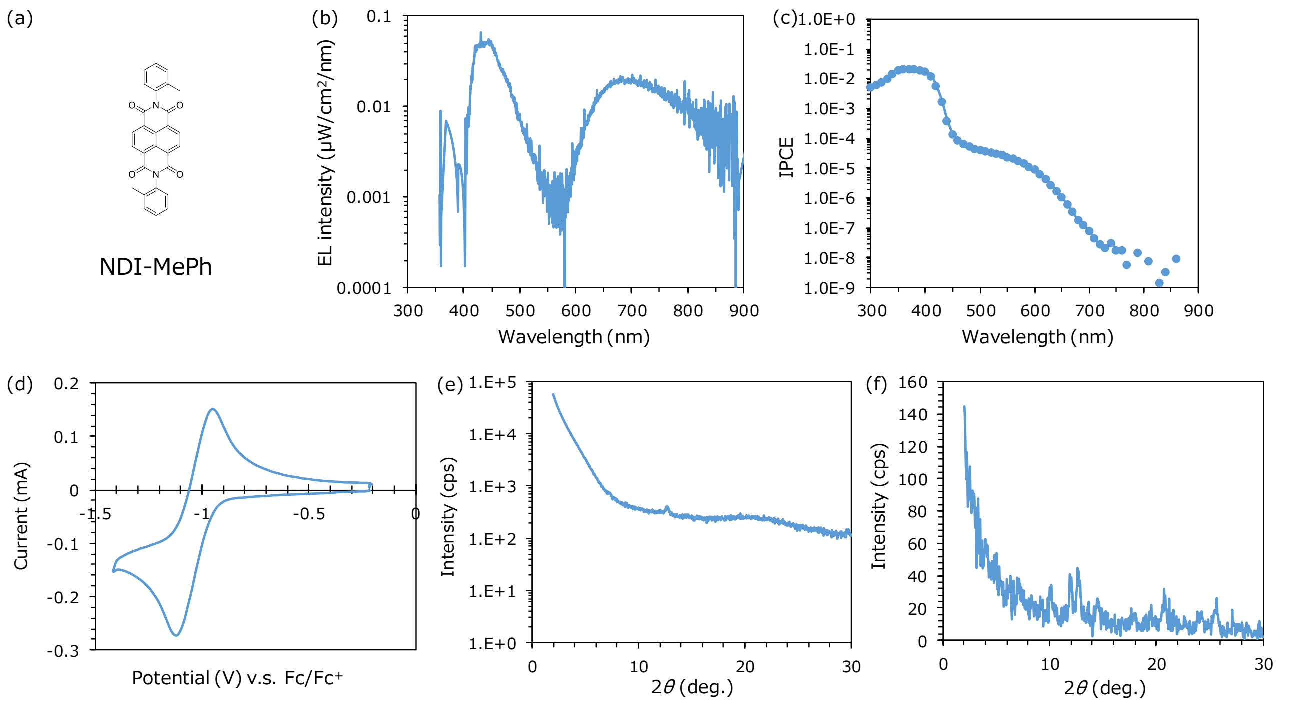

Figure S20. (a) Chemical structure of NDI-MePh. (b) EL emission spectrum under a constant current flow (100 mA/cm^2) and (c) highly sensitive IPCE spectra of the 1,2-ADN/NDI-MePh device. (d) Cyclic voltammogram of NDI-MePh in solution. (e) Out-of-plane and (f) in-plane XRD patterns of the NDI-MePh film on a silicon wafer.

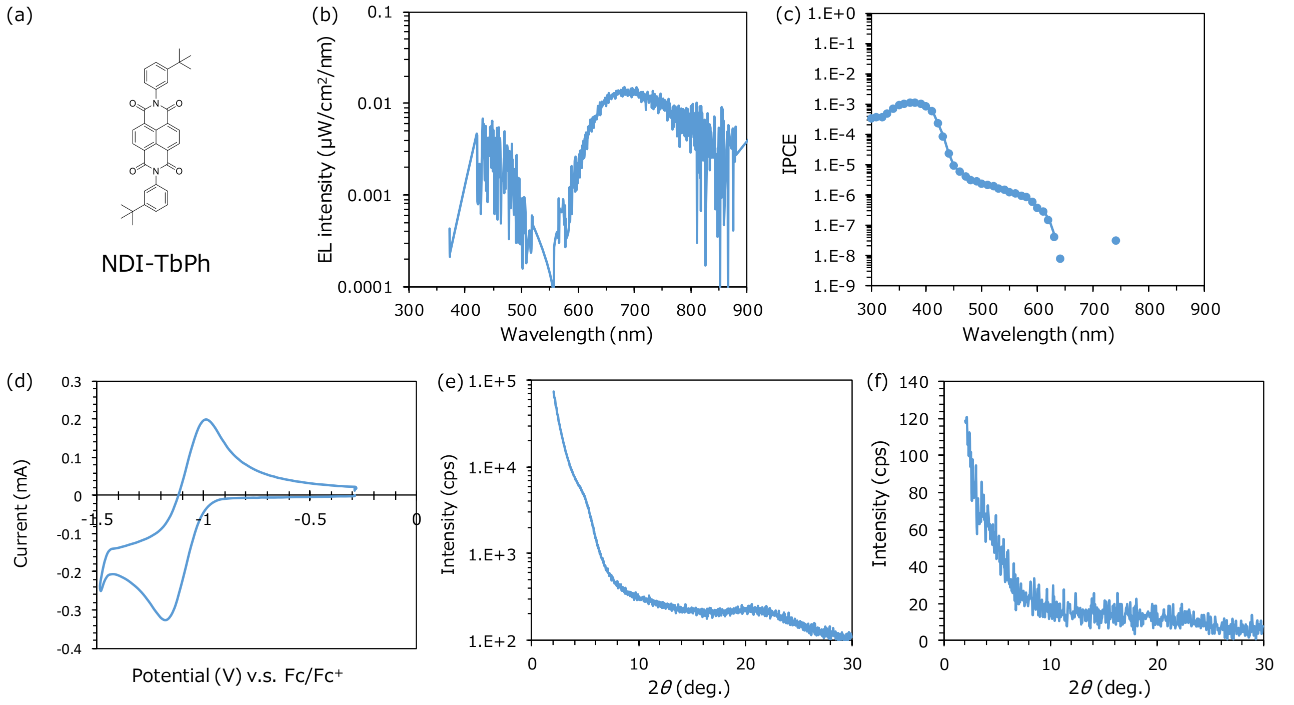

Figure S21. (a) Chemical structure of NDI-TbPh. (b) EL emission spectrum under a constant current flow (100 mA/cm^2) and (c) highly sensitive IPCE spectra of the 1,2-ADN/NDI-TbPh device. (d) Cyclic voltammogram of NDI-TbPh in solution. (e) Out-of-plane and (f) in-plane XRD patterns of the NDI-TbPh film on a silicon wafer.

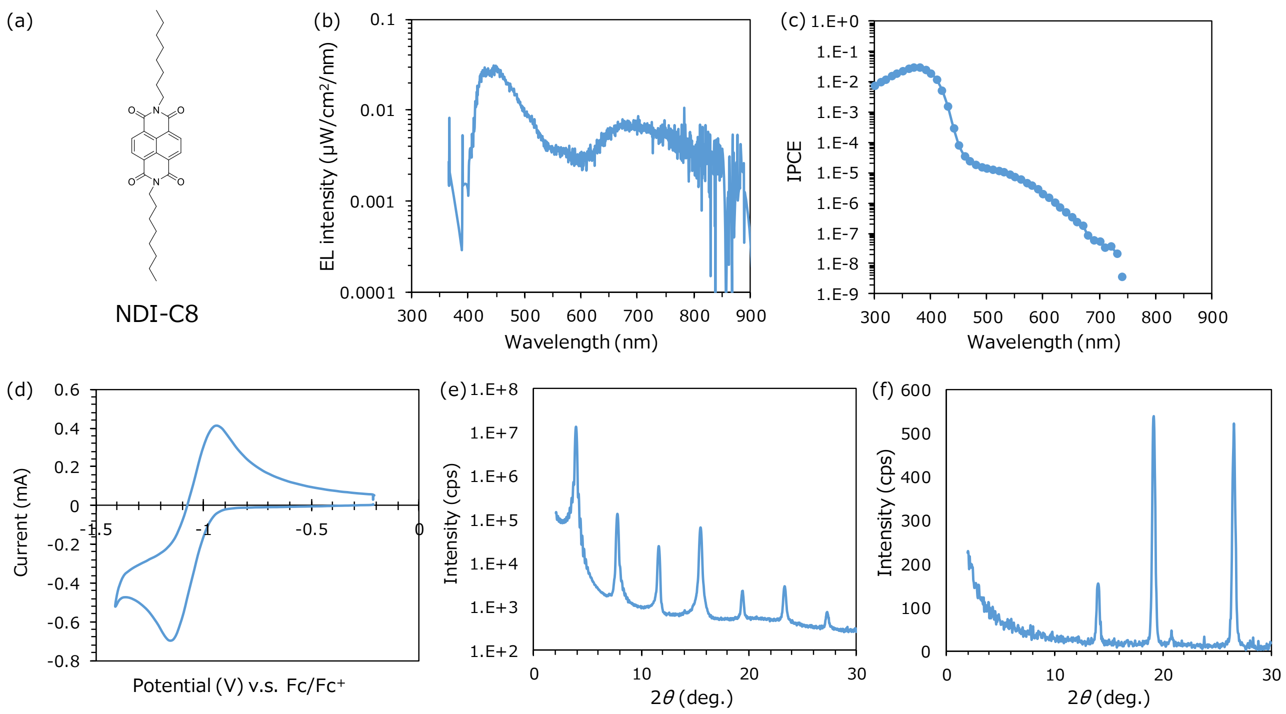

Figure S22. (a) Chemical structure of NDI-C8. (b) EL emission spectrum under a constant current flow (100 mA/cm^2) and (c) highly sensitive IPCE spectra of the 1,2-ADN/NDI-C8 device. (d) Cyclic voltammogram of NDI-C8 in solution. (e) Out-of-plane and (f) in-plane XRD patterns of the NDI-C8 film on a silicon wafer.

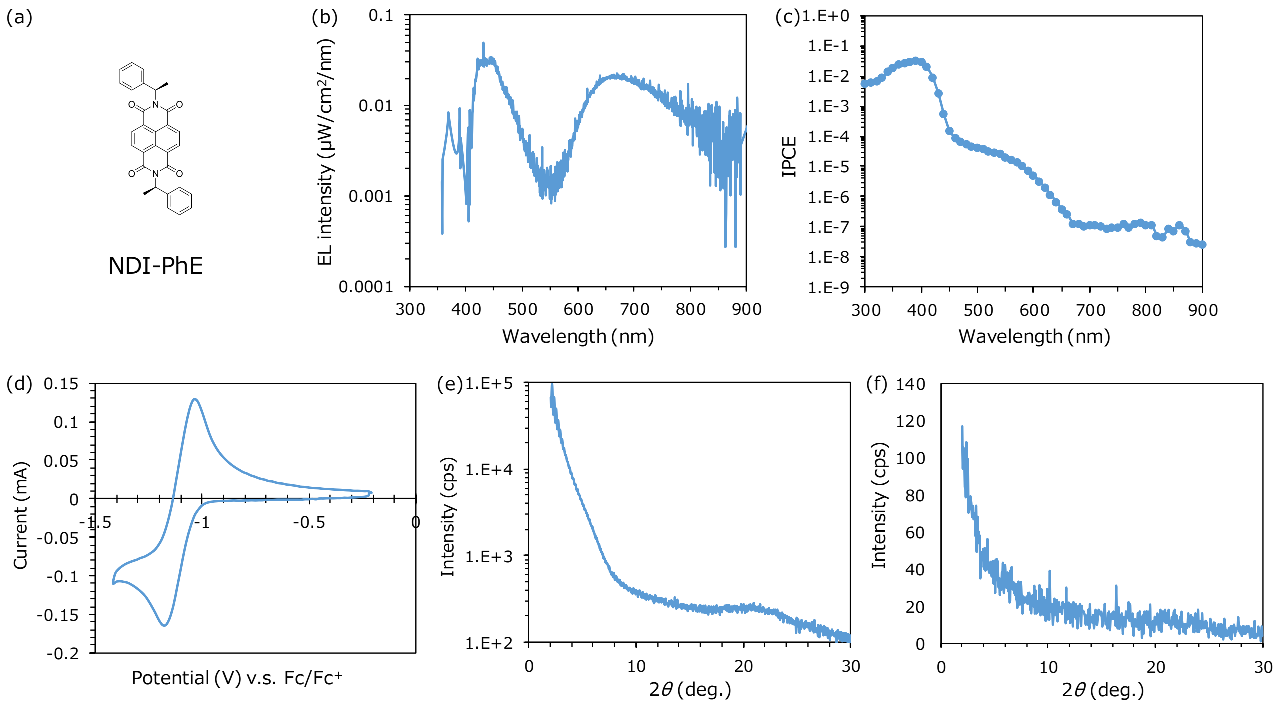

Figure S23. (a) Chemical structure of NDI-PhE. (b) EL emission spectrum under a constant current flow (100 mA/cm^2) and (c) highly sensitive IPCE spectra of the 1,2-ADN/NDI-PhE device. (d) Cyclic voltammogram of NDI-PhE in solution. (e) Out-of-plane and (f) in-plane XRD patterns of the NDI-PhE film on a silicon wafer.

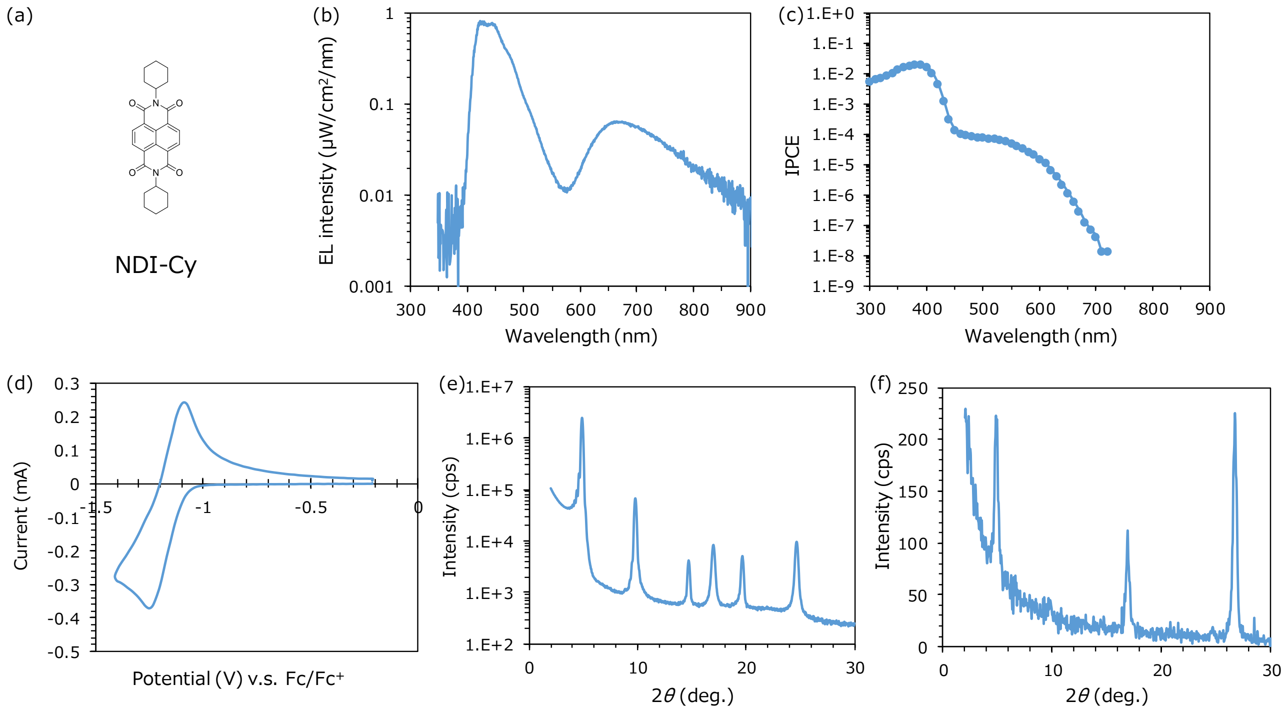

Figure S24. (a) Chemical structure of NDI-Cy. (b) EL emission spectrum under a constant current flow (100 mA/cm^2) and (c) highly sensitive IPCE spectra of the 1,2-ADN/NDI-Cy device. (d) Cyclic voltammogram of NDI-Cy in solution. (e) Out-of-plane and (f) in-plane XRD patterns of the NDI-Cy film on a silicon wafer.

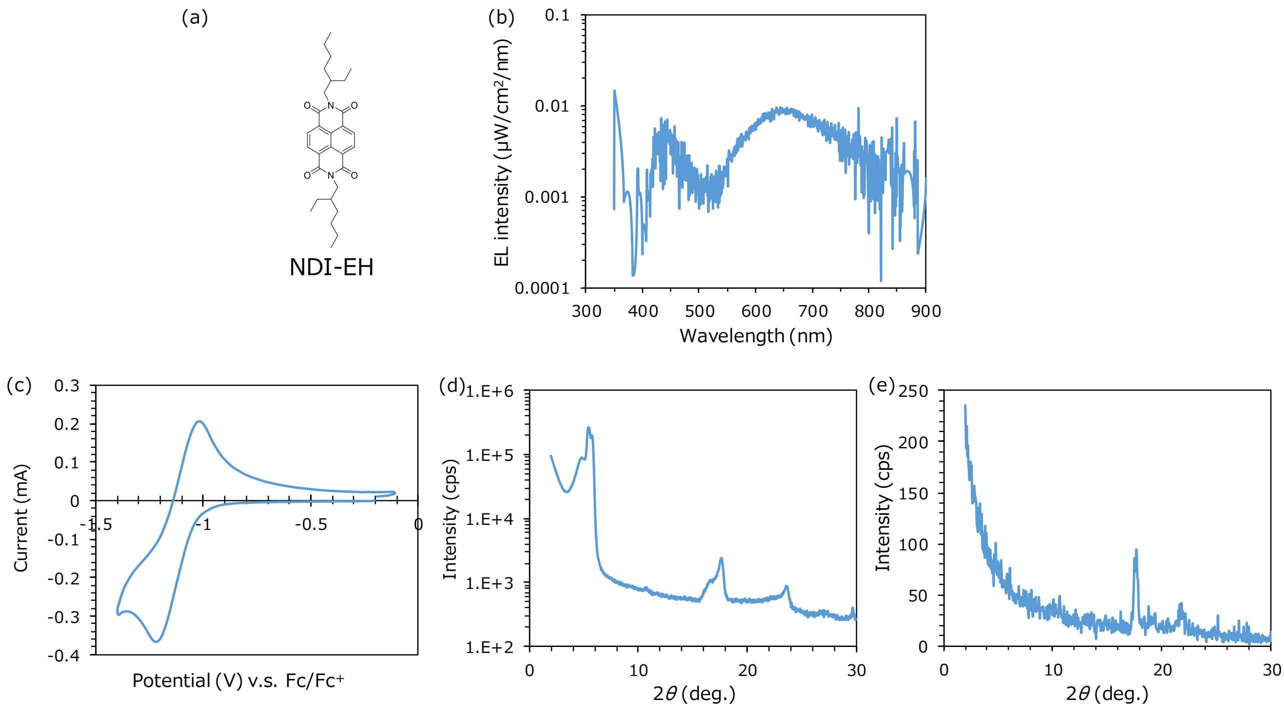

Figure S25. (a) Chemical structure of NDI-EH. (b) EL emission spectrum under a constant current flow (100 mA/cm^2) of the 1,2-ADN/NDI-EH device. (c) Cyclic voltammogram of NDI-EH in solution. (d) Out-of-plane and (e) in-plane XRD patterns of the NDI-EH film on a silicon wafer.

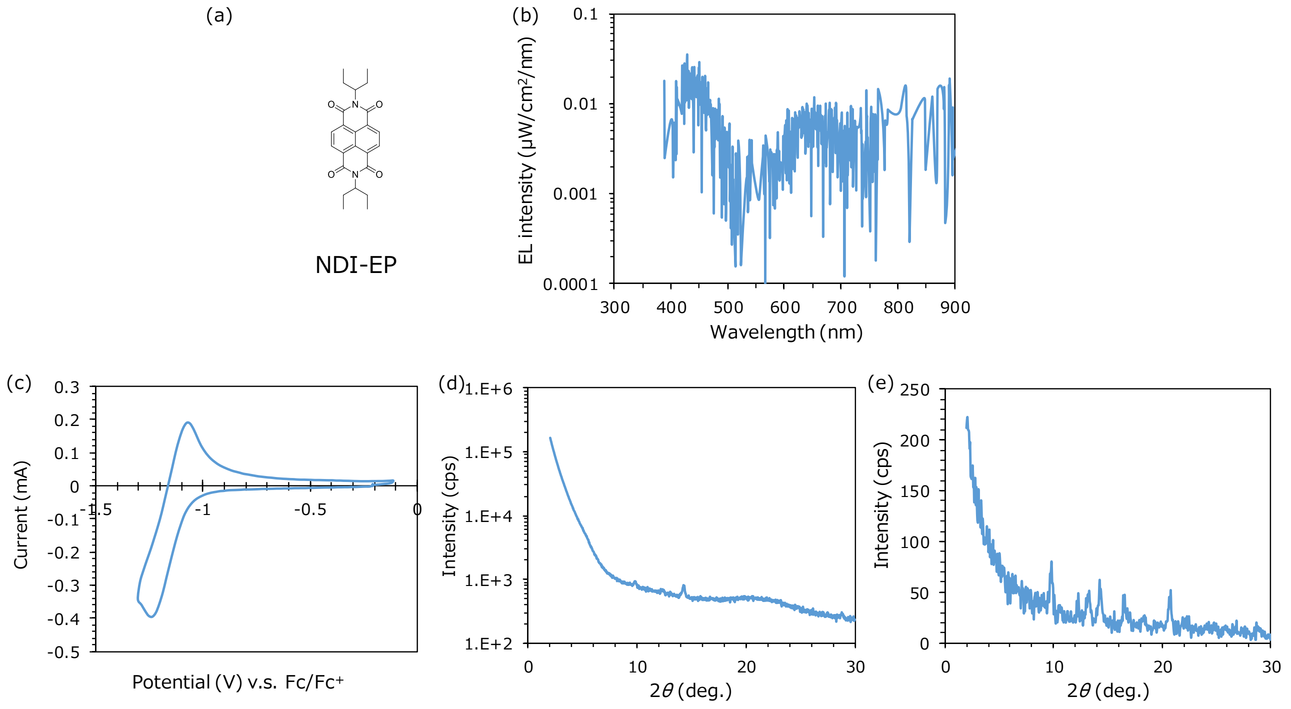

Figure S26. (a) Chemical structure of NDI-EP. (b) EL emission spectrum under a constant current flow (100 mA/cm^2) of the 1,2-ADN/NDI-EP device. (c) Cyclic voltammogram of NDI-EP in solution. (d) Out-of-plane and (e) in-plane XRD patterns of the NDI-FP film on a silicon wafer.

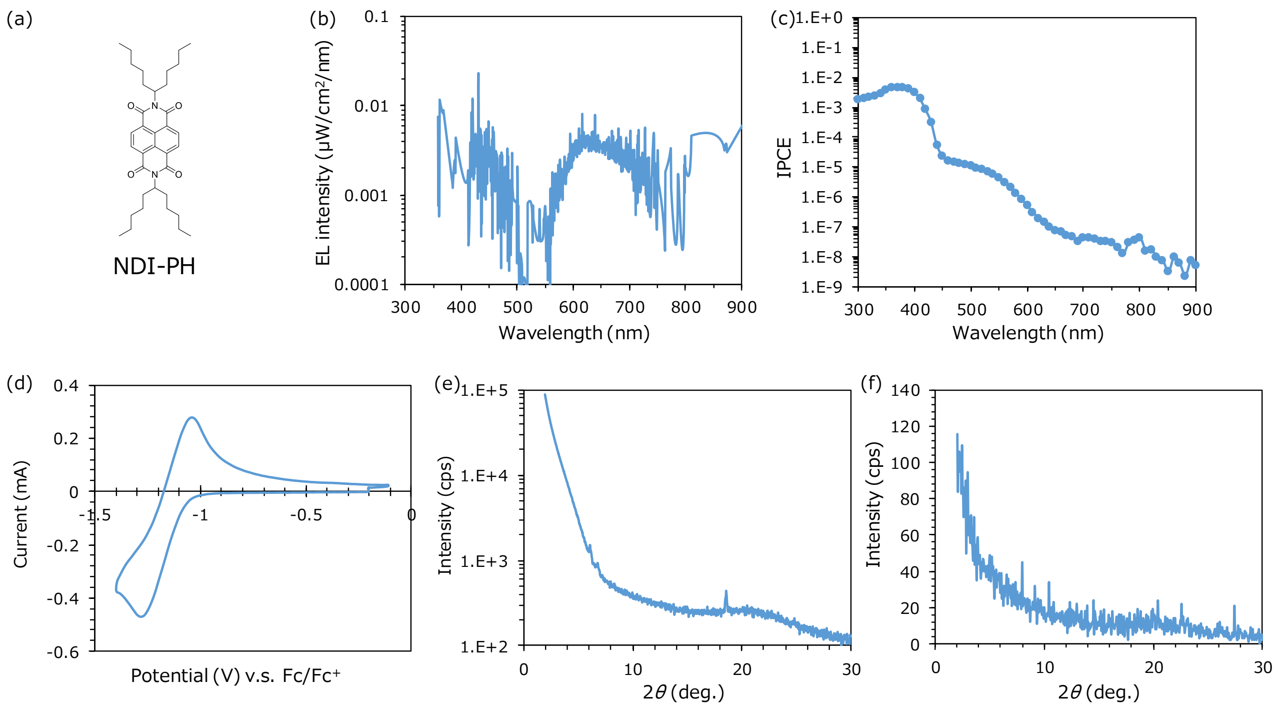

Figure S27. (a) Chemical structure of NDI-PH. (b) EL emission spectrum under a constant current flow (100 mA/cm^2) and (c) highly sensitive IPCE spectra of the 1,2-ADN/NDI-PH device. (d) Cyclic voltammogram of NDI-PH in solution. (e) Out-of-plane and (f) in-plane XRD patterns of the NDI-PH film on a silicon wafer.

Table S1. LUMO energy levels of NDI-derivatives versus vacuum level measured by CV in solution. The redox potential of ferrocene versus vacuum level is assumed as -4.80 eV. CV of NDI-HF cannot be measured because of its low solubility.

Name of NDI derivatives	Groups	LUMO energy levels (eV)
FPh	Aryl	-3.83
Naph	Aryl	-3.78
MePh	Aryl	-3.76
BPh	Aryl	-3.76
Ph	Aryl	-3.76
C8	Alkyl	-3.75
MeF	Aryl	-3.74
TbPh	Aryl	-3.71
PhE	Alkyl	-3.69
EH	Alkyl	-3.67
EP	Alkyl	-3.64
Cy	Alkyl	-3.63
PH	Alkyl	-3.63

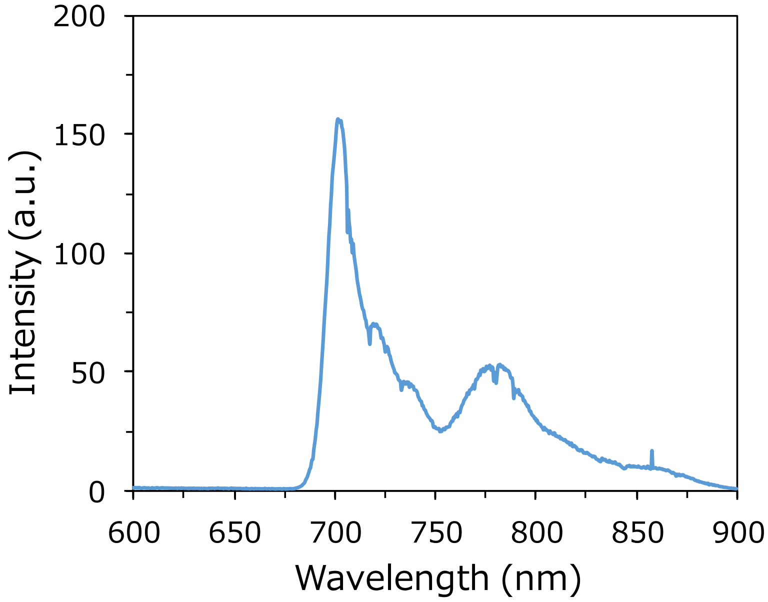

Figure S28. Phosphorescent spectrum of 1,2-ADN in chloroform solution at 77 K. Chopping frequency is 25 ms and integration period is from 1 ms to 6 ms. Phosphorescent signal could not be obtained from the thin film of 1,2-ADN.

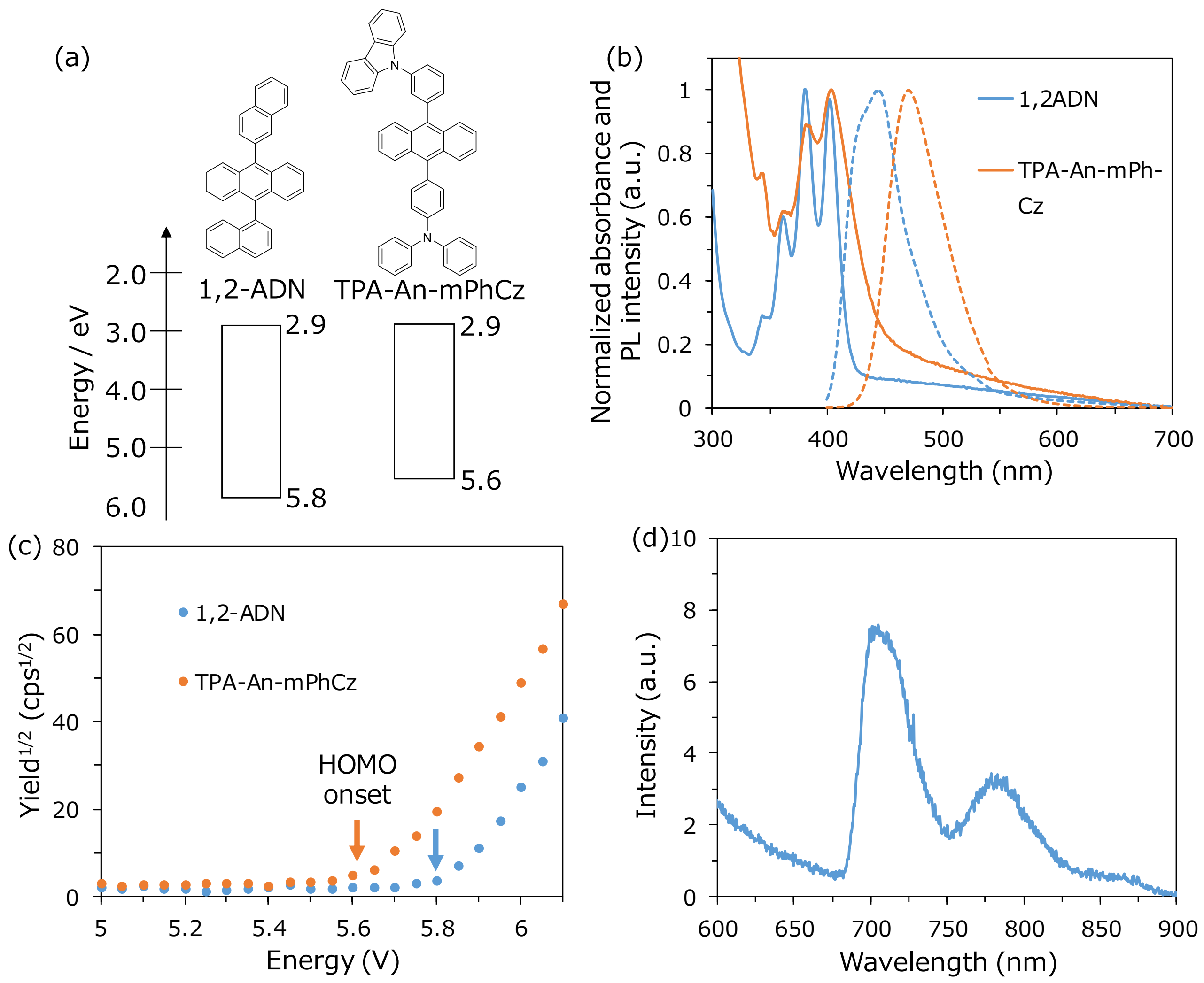

Figure S29. (a) Chemical structures and energy levels of 1,2-ADN and TPA-An-mPhCz. (b) Normalized absorbance (solid line) and PL emission (broken line) of 1,2-ADN and TPA-An-mPhCz thin film. (c) PYS of 1,2-ADN and TPA-An-mPhCz thin film. (d) Phosphorescent spectrum of TPA-An-mPhCz in chloroform solution at 77 K.

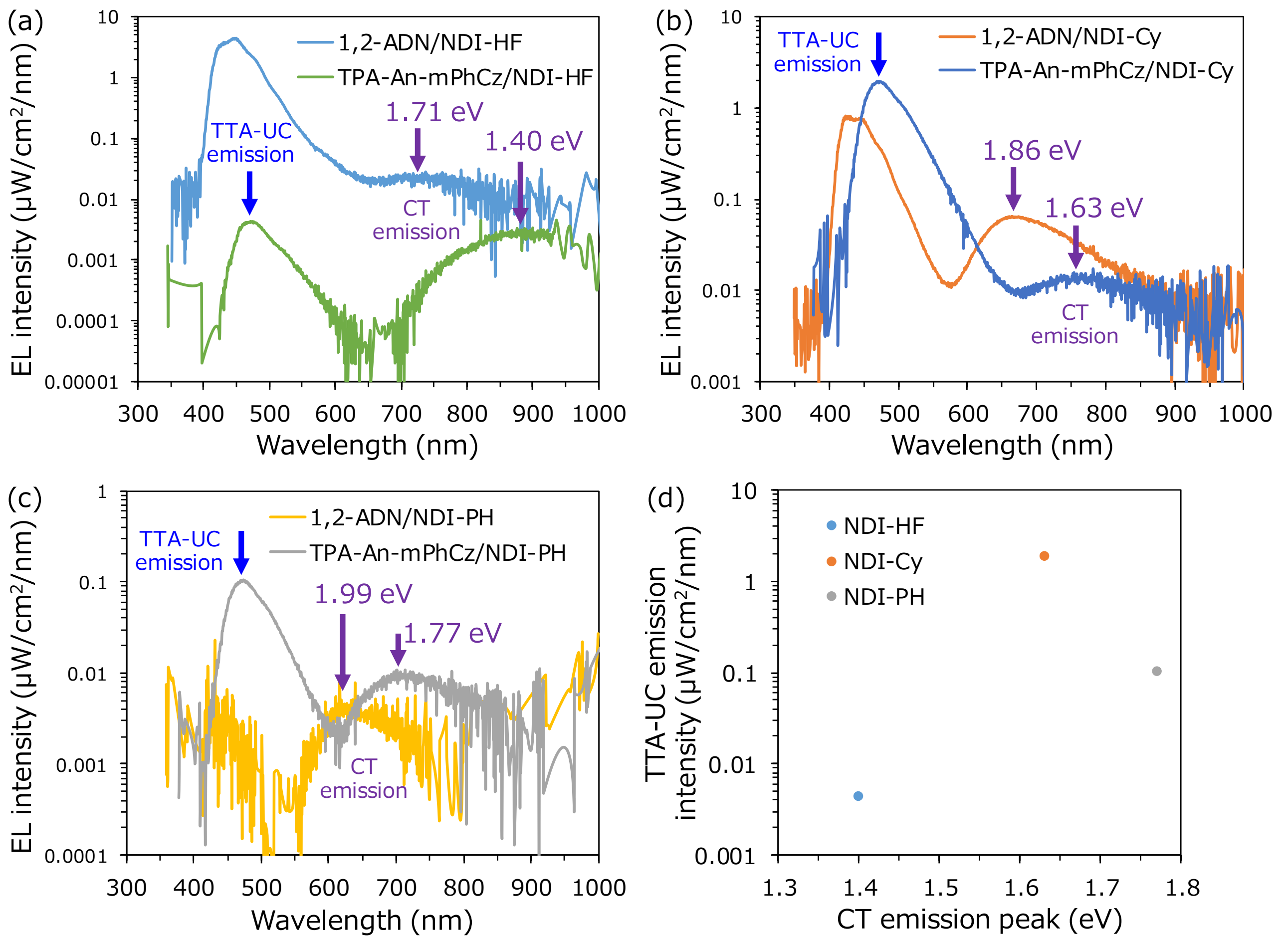

Figure S30. EL emission spectra of (a) 1,2-ADN or TPA-An-mPhCz/NDI-HF, (b) 1,2-ADN or TPA-An-mPhCz/NDI-Cy, and (c) 1,2-ADN or TPA-An-mPhCz/NDI-PH devices under a constant current flow (100 mA/cm^2). (d) Plots of TTA-UC emission (blue arrow in Figure S29a~c) intensity versus energy of CT emission peak for the devices with TPA-An-mPhCz and NDI derivatives.

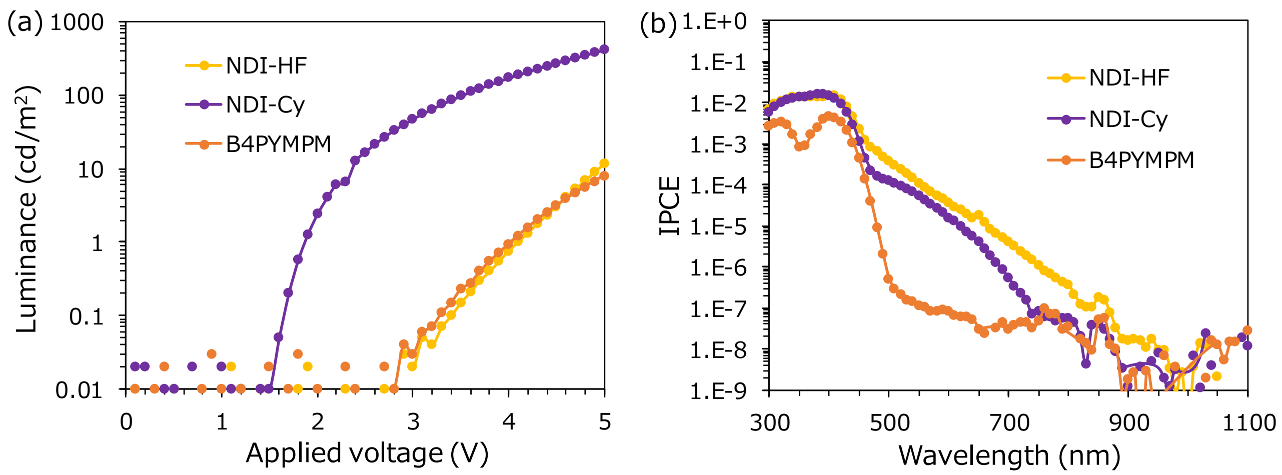

Figure S31. (a) *L–V* curves for the TPA-An-mPhCz/NDI-HF (yellow), TPA-An-mPhCz/NDI-Cy (purple) or TPA-An-mPhCz/B4PYMPM (orange) devices. (b) Highly sensitive IPCE spectra.

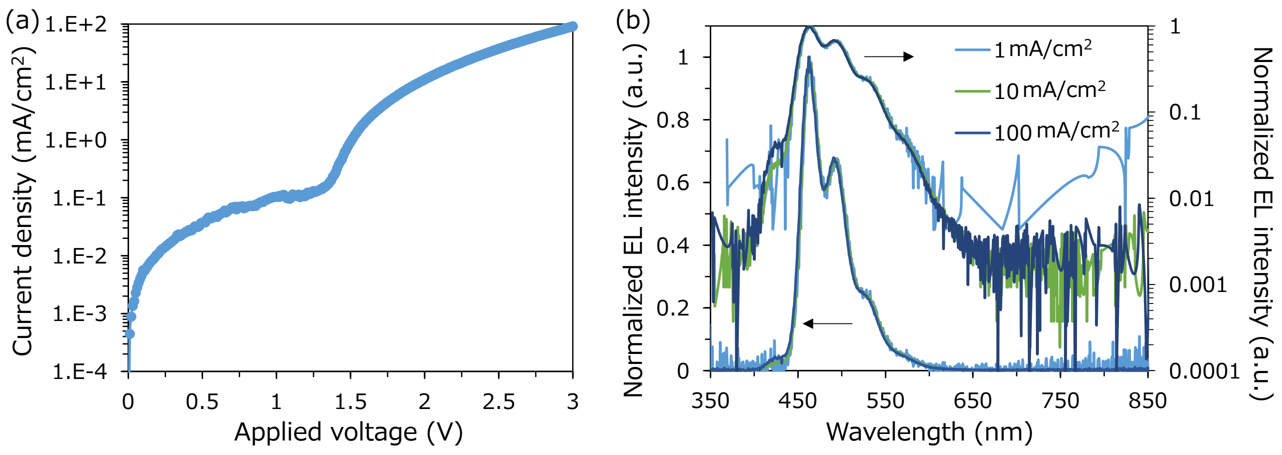

Figure S32. (a) *J–V* curve of the TbPe-doped device. (b) EL spectra of the TbPe-doped device at different current densities.

Table S2. Summary of the references of a blue LED with low turn-on voltages used in Figure 4e. Types of the device, host/dopant materials in the emission layer (EML), peak wavelength of emissio, turn-on voltage, maximum EQE, maximum luminance, Commission Internationale de l'Éclairage coordinates (CIE), and reference number are listed. The references are summarized below. All the references of OLED use a device structure with ITO as an anode and a bottom emission.

Types	Host/Dopant in EML	Peak (nm)	Turn-on (V)	Max. EQE (%)	Max. Luminance (cd/m^2)	CIE	Ref.
UC-OLED	1,2-ADN/TbPe	468	1.47	1.37	>1000	0.14, 0.21	This work
Fluorescent	1,2-ADN/BD-1	464	2.6	9.3	>10000	-	1
Fluorescent	1,2-ADN/ν-DABNA	471	2.8	7.0	>10000	0.12, 0.13	2
Fluorescent	Pyrene derivative/DSA-Ph	468	2.6	6.84	>10000	0.15, 0.28	3

Phosphorescent	CbBPCb/FIrpic	473	3.34	30	>1000	-	4
Phosphorescent	PyTzSCz/FIrpic	469	2.6	26.4	>10000	0.14, 0.27	5
Phosphorescent	SPPI-C2Py/FIrpic	471	2.5	22.43	>10000	0.15, 0.37	6
Phosphorescent	mCP:B3PYMPM/Firpic	472	2.6	23.3	>10000	-	7
TADF	DBFPO:TSPO1/TDBA-DI	458	2.5	41.2	>10000	-	8
TADF	DPEPO/DspiroS-TRZ	480	2.9	38.4	>10000	0.18, 0.37	9
TADF	HDT-1/ν-DABNA	470	3	27	>10000	0.13, 0.16	10
TADF	BIZ-2Me-TRZ/DtBuCzB	489	2.9	28.4	>1000	0.09, 0.40	11
Inorganic	InGaN	455	2.4	40.94	>10000	-	12

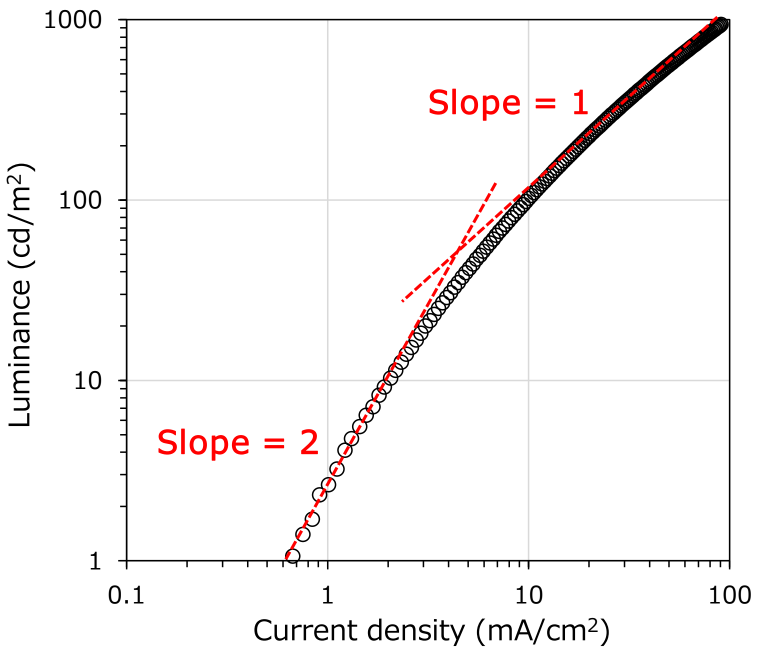

Figure S33. *L-J* curve of the TbPe-doped device.

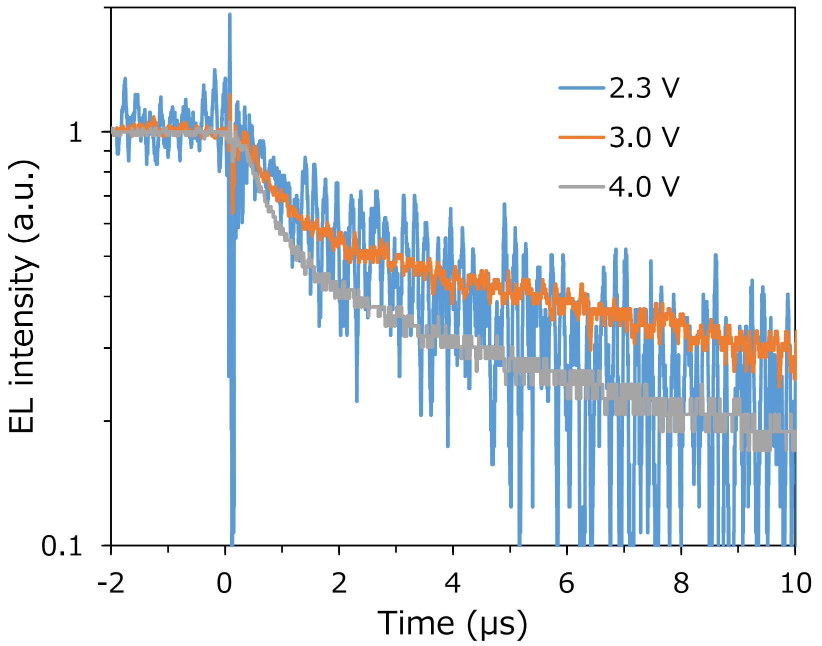

Figure S34. Decay dynamics of EL emission of the TbPe-doped device. 2.3 V (blue), 3.0 V (orange) and 4.0 V (gray) were applied to the device.

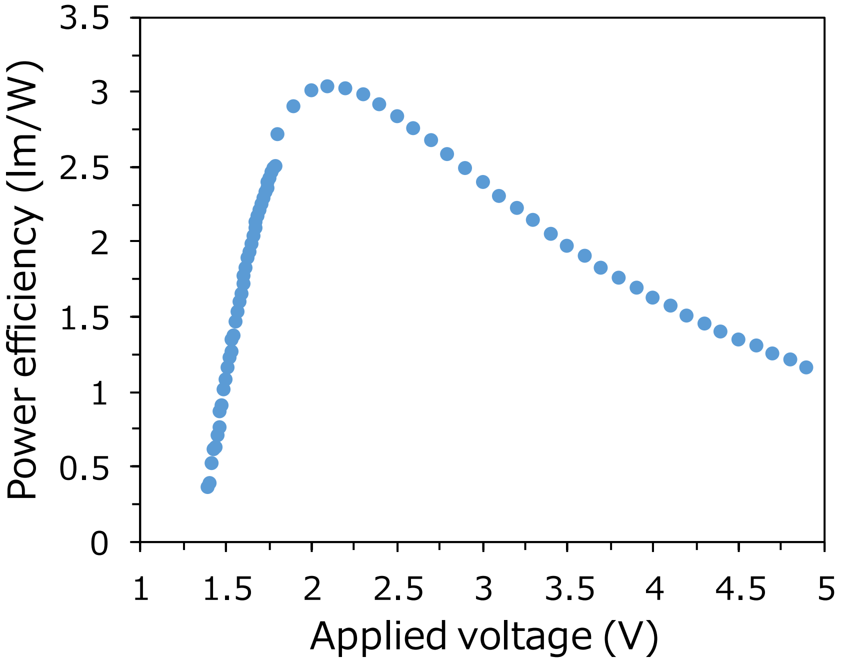

Figure S35. Power efficiency of the TbPe-doped device.

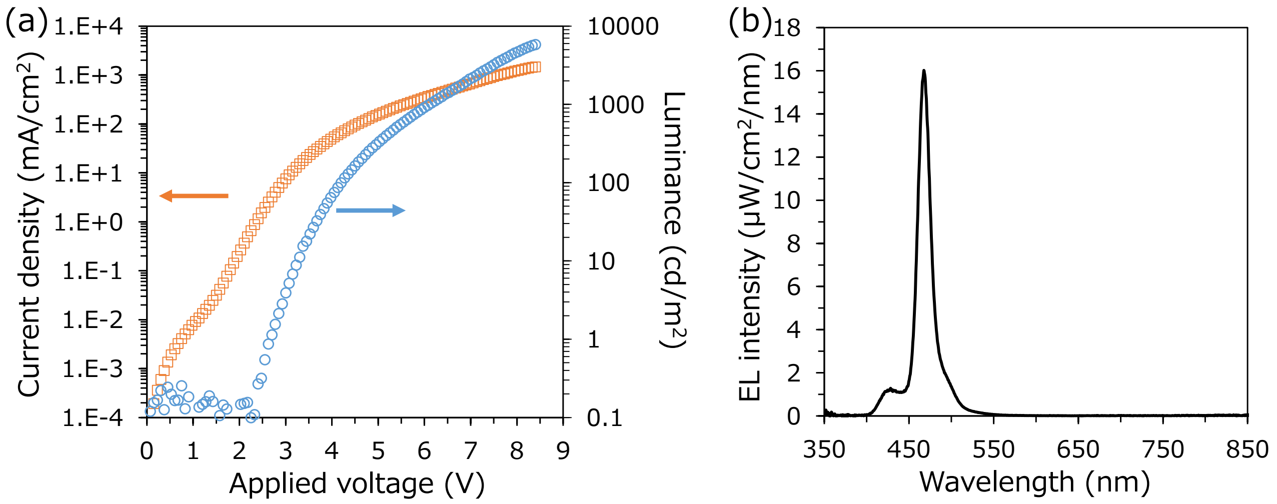

Figure S36. (a) *J*–*V* (orange square) and *L*–*V* (blue circle) curves for the ν-DABNA-doped device. (b) EL emission spectrum of the ν-DABNA-doped 1,2-ADN/NDI-HF device under a constant current flow (100 mA/cm^2).

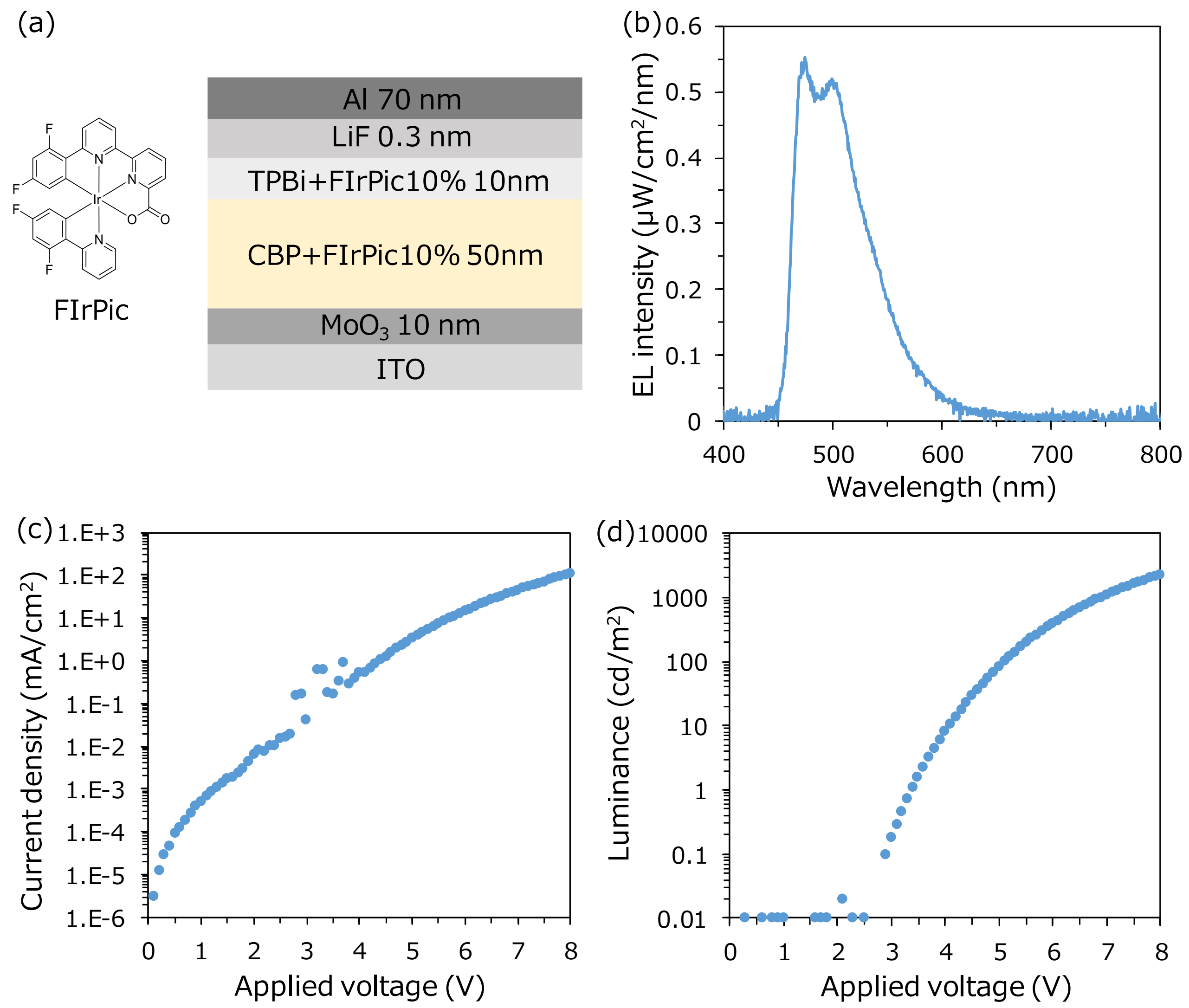

Figure S37. (a) Chemical structure of FIrPic and the device structure. (b) EL emission spectra of the FIrPic-doped phosphorescent device under a constant current flow (100 mA/cm^2). (c) *J*–*V* and (d) *L*–*V* curve of FIrPic-doped phosphorescent device.

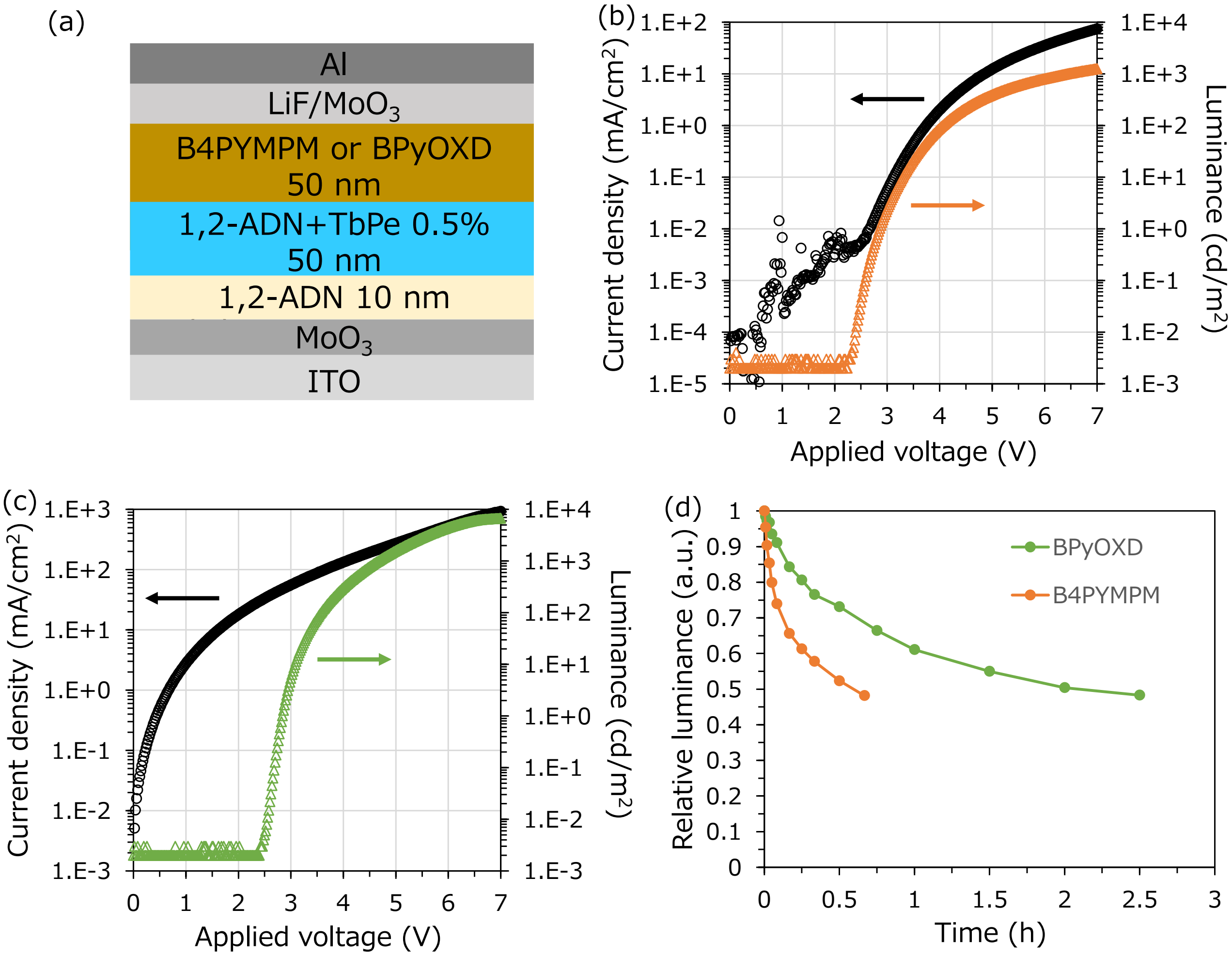

Figure S38. (a) The schematic structure of the TbPe-doped device with B4PYMPM or BPyOXD as the electron transport layer. *J*–*V* (circle) and *L*–*V* (triangle) curves for the TbPe-doped device with (b) B4PYMPM or (c) BPyOXD. (d) The operation lifetime measurement under the initial luminance condition at 1000 cd/m^2 of the TbPe-doped device with B4PYMPM (orange) and BPyOXD (green).

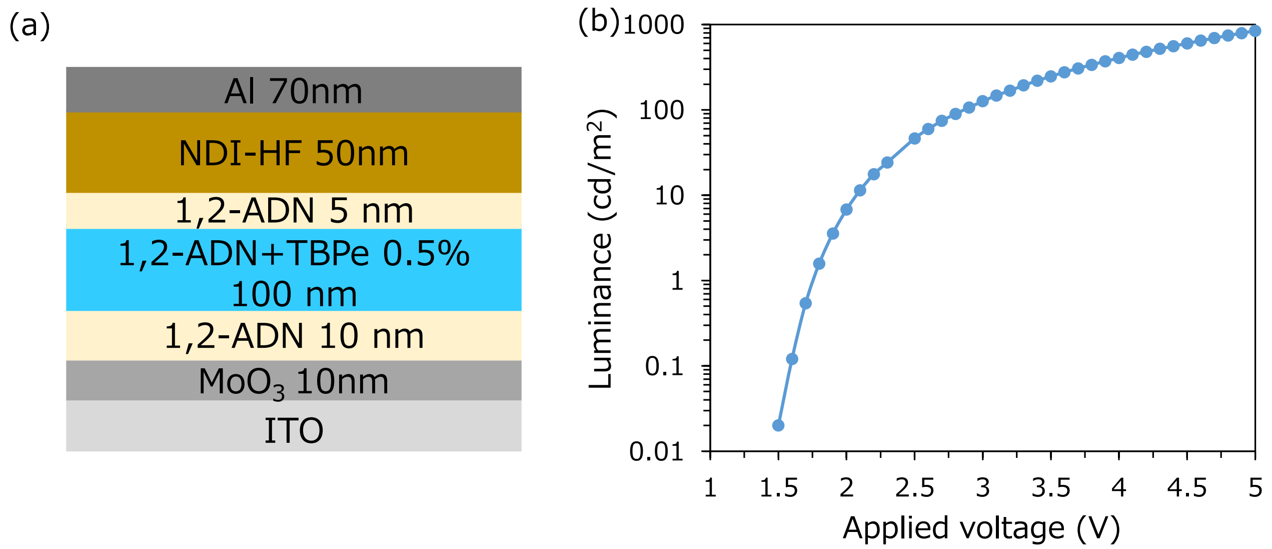

Figure S39. (a) The device structure of the UC-OLED without LiF as electron injection layer. (b) *L*–*V* curve of the UC-OLED without LiF.

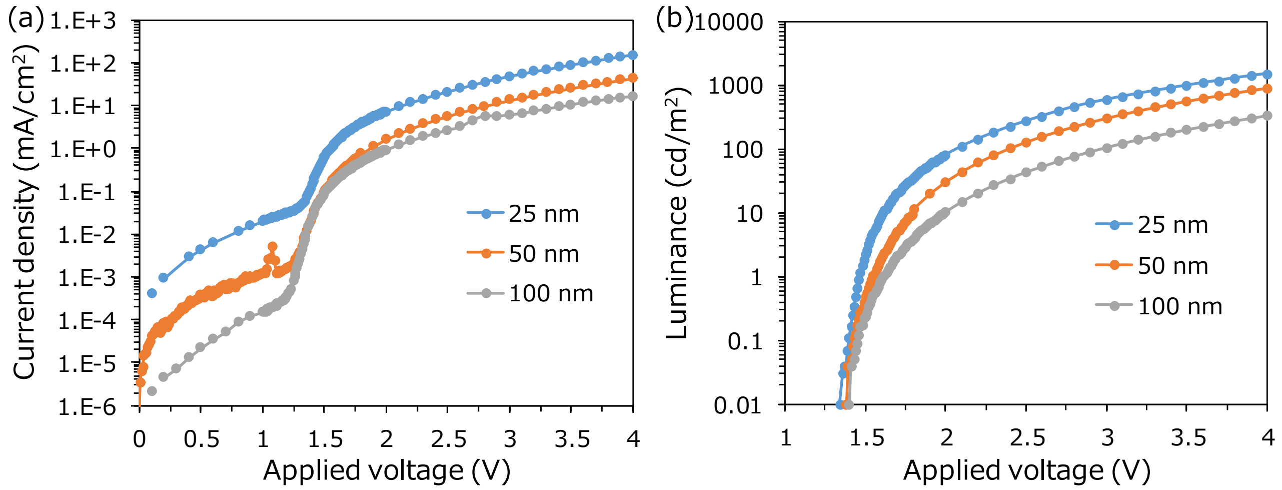

Figure S40. (a) *J*–*V* and (b) *L*–*V* curves of the device with the different TbPe-doped layer thickness of 25 nm (blue), 50 nm (orange) and 100 nm (grey).

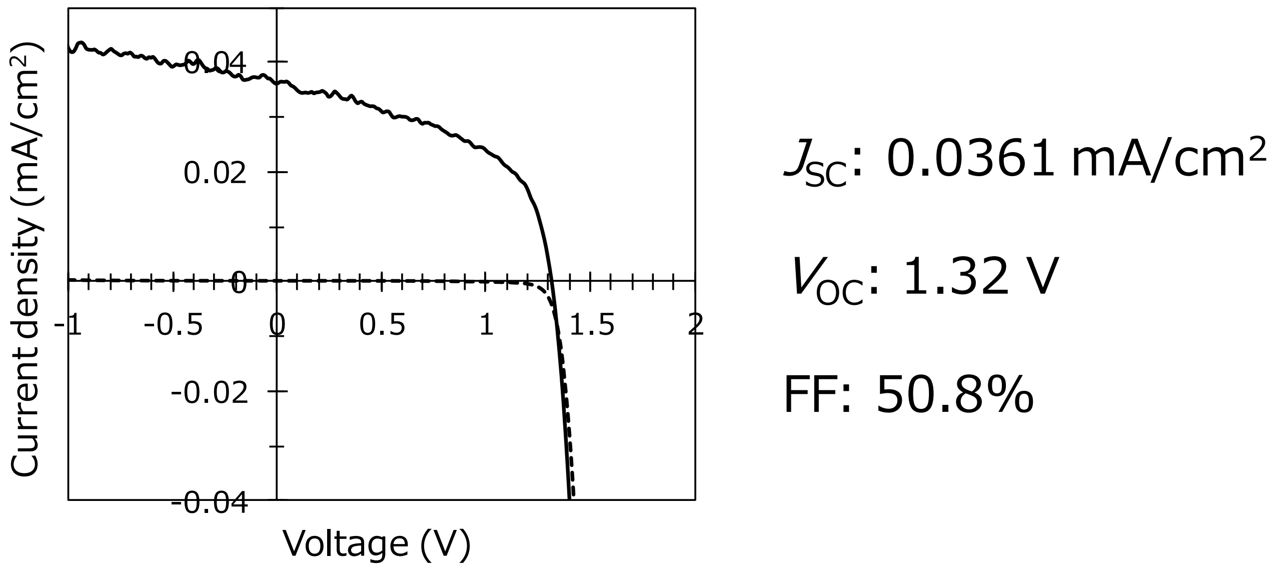

Figure S41. The diode characteristic of the UC-OLED under dark (broken line) and AM 1.5, 100 mW/cm^2 irradiation (solid line).

Supplementary References

1 Sasaki, T. *et al.* Unravelling the electron injection/transport mechanism in organic light-emitting diodes. *Nat Commun* **12**, 2706, doi:10.1038/s41467-021-23067-2 (2021).

2 Nguyen, T. B., Nakanotani, H., Chan, C. Y., Kakumachi, S. & Adachi, C. Enhancing Triplet-Triplet Upconversion Efficiency and Operational Lifetime in Blue Organic Light-Emitting Diodes by Utilizing Thermally Activated Delayed Fluorescence Materials. *ACS Appl Mater Interfaces* **15**, 23557-23563, doi:10.1021/acsami.3c02855 (2023).

3 Ran, H. *et al.* Blue-emitting butterfly-shaped donor–acceptor-type 1,3,5,9-tetraarylpyrenes: easily available, low-cost conventional fluorophores for high-performance near ultraviolet electroluminescence with CIEy < 0.05. *J Mater Chem C* **9**, 260-269, doi:10.1039/d0tc03612a (2021).

4 Lee, C. W. & Lee, J. Y. Above 30% external quantum efficiency in blue phosphorescent organic light-emitting diodes using pyrido[2,3-b]indole derivatives as host materials. *Adv Mater* **25**, 5450-5454, doi:10.1002/adma.201301091 (2013).

5 Liu, D., Wang, F. & Yao, R. Molecular evolution of host materials by regular tuning of n/p ratio for high-performance phosphorescent organic light-emitting diodes. *J Mater Chem C* **6**, 7839-7846, doi:10.1039/c8tc02432d (2018).

6 Jayabharathi, J., Sivaraj, S., Thanikachalam, V. & Anudeebhana, J. Multifunctional pyridine styrylphenanthroimidazoles: electron transport materials for blue FOLEDs with low efficiency roll-off and hosts for PHOLEDs with low turn-on voltage. *J Mater Chem C* **9**, 10334-10346,

doi:10.1039/d1tc02131a (2021).
7 Xie, J. *et al.* Improvement of exciton utilization by suppressing exciton leakage for high efficiency blue and white organic light-emitting diodes. *J Mater Chem C* **10**, 8349-8355, doi:10.1039/d2tc00636g (2022).
8 Vasilopoulou, M. *et al.* High efficiency blue organic light-emitting diodes with below-bandgap electroluminescence. *Nat Commun* **12**, 4868, doi:10.1038/s41467-021-25135-z (2021).
9 Li, W. *et al.* Spiral Donor Design Strategy for Blue Thermally Activated Delayed Fluorescence Emitters. *ACS Appl Mater Interfaces* **13**, 5302-5311, doi:10.1021/acsami.0c19302 (2021).
10 Chan, C.-Y. *et al.* Stable pure-blue hyperfluorescence organic light-emitting diodes with high-efficiency and narrow emission. *Nat Photon* **15**, 203-207, doi:10.1038/s41566-020-00745-z (2021).
11 Wu, Y. *et al.* Benzimidazole-triazine based n-type hosts with twisted structure and high triplet energy level for efficient blue narrowband emitting OLEDs. *Chem Eng J* **465**, 142848, doi:10.1016/j.cej.2023.142848 (2023).
12 Weng, Y. *et al.* Hybrid Device of Blue GaN Light-Emitting Diodes and Organic Light-Emitting Diodes with Color Tunability for Smart Lighting Sources. *ACS Omega* **7**, 5502-5509, doi:10.1021/acsomega.1c06934 (2022).
